# Supplementary material for: Understanding the implementation and adoption of an information technology intervention to support medicine optimisation in primary care: qualitative study using strong structuration theory
Source: BMJ Open. 2017 May 10;7(5):e014810. doi: 10.1136/bmjopen-2016-014810 (PMC5736096; doi:10.1136/bmjopen-2016-014810)
Supplement: Supplementary data [file bmjopen-2016-014810supp001.docx]

**Understanding the implementation and adoption of an information technology intervention to support medicines optimisation in primary care: qualitative study using strong structuration theory**

**COREQ checklist**

Note: in order to minimize the length of the manuscript, some of the details on the checklist (marked ‘*’) are not included in the manuscript.

| *Guide question* | | *Response* | *Page number in manuscript* |
| --- | --- | --- | --- |
| 1 | Interviewer/facilitator | MJ conducted the interviews. MJ and RLH facilitated the focus groups | 9 |
| 2 | Credentials | MJ holds an MSc in Health Psychology  RLH holds a PhD in Medic8ines safety in Primary care | 9 |
| 3 | Occupation | MJ : Research Associate in medication safety;  RLH: freelance research pharmacist | 9 |
| 4 | Gender | MJ male; RLH female | 9 |
| 5 | Experience and training | Both researchers have previous experience of undertaking qualitative research in healthcare at PhD and postdoctoral level | 9 |
| 6 | Relationship established | The researchers were not known to the participants prior to the study | 9 |
| 7 | Participant knowledge of the researcher | Participants were made aware of the reasons for doing the research via the information which was sent to the participant prior to the interview | * |
| 8 | Researcher characteristics | The researchers had identified the study topic as part of larger programmes of work in their research groups, medication safety in primary care. | 9 |
| 9 | Methodological orientation and theory | Strong structuration theory. The analysis was thematic using template analysis. | 5-6, 10 |
| 10 | Sampling | Individual participants were recruited on a purposive basis via the study CCG or through community pharmacy networks. All participants were chosen to fit the sampling frame (people within the CCG's geographical area who represented the stakeholder groups: pharmacists, doctors, general practice managers and patients) | 8-9 |
| 11 | Method of approach | Participants were approached by telephone or email | 9 |
| 12 | Sample size | 19 participants | 8-9 |
| 13 | Non-participation | A number of possible participants were approached but declined to participate. Predominantly this was for reasons of time, workload or lack of use of the system. These included 2 pharmacist technicians, 2 GPs, 2 community pharmacists and 8 general practice managers. | * |
| 14 | Setting of data collection | Four interviews were conducted by telephone and one at the CCG offices, the focus groups were conducted at the CCG offices or at a local hotel. | 9 |
| 15 | Presence of non-participants | No non-participants were present | 9 |
| 16 | Description of sample | See Table 1 of the main manuscript | 8 |
| 17 | Interview guide | In the interviews and focus groups we explored experiences of working with the EMOS, perceptions of the system, benefits and drawbacks, the organisational structures and roles required for its use and the circumstances under which it was considered most effective. No pilot testing was undertaken due to the small scale nature of the study, the timescale of the study and the difficulties of recruitment. | 9 |
| 18 | Repeat interviews | None -One GP was interviewed and participated in a focus group | 9 |
| 19 | Audio/visual recording | Audio recording only, with consent from the participant | 9 |
| 20 | Field notes | None | * |
| 21 | Duration | The interviews lasted between 20 and 50 mins. The focus groups lasted between 57-112 mins. | 9 |
| 22 | Data saturation | Data collection continued until saturation was reached and no new themes emerged from the interviews and focus groups. | 9 |
| 23 | Transcripts returned | No transcripts were returned to participants | * |
| 24 | Number of data coders | MJ coded the data but regular discussions codes were held with all authors. Coding template reviewed by MJ and DLP | 10 |
| 25 | Description of the coding tree | A coding tree description is not given but details on a priori codes is included | 10 |
| 26 | Derivation of themes | A priori thematic codes were applied to the data and new themes emerged from the data. This is described in the analysis section | 10 |
| 27 | Software | QSRNvivo 10 software was utilised to manage the data | 10 |
| 28 | Participant checking | No | * |
| 29 | Quotations presented | Please see the results section of the manuscript | 10-17 |
| 30 | Data and findings consistent |  | 10-17 |
| 31 | Clarity of major themes |  | 10-17 |
| 32 | Clarity of minor themes |  | 10-17 |
