# Supplementary material for: Understanding the implementation and adoption of an information technology intervention to support medicine optimisation in primary care: qualitative study using strong structuration theory
Source: BMJ Open. 2017 May 10;7(5):e014810. doi: 10.1136/bmjopen-2016-014810 (PMC5736096; doi:10.1136/bmjopen-2016-014810)
Supplement: Author's manuscript [file bmjopen-2016-014810.draft_revisions.pdf]

BMJ Open

Understanding the implementation and adoption of an information technology intervention to support medicines optimisation in primary care: qualitative study using strong structuration theory

Journal:	*BMJ Open*
Manuscript ID	bmjopen-2016-014810
Article Type:	Research
Date Submitted by the Author:	25-Oct-2016
Complete List of Authors:	Jeffries, Mark; University of Manchester, Centre for Pharmacoepidemiology and Drug Safety,School of Health Sciences; University of Manchester, NIHR Greater Manchester Primary Care Patient Safety Translational Research Centre<br>Phipps, Denham; University of Manchester, Centre for Pharmacoepidemiology and Drug Safety, School of Health Sciences; University of Manchester, NIHR Greater Manchester Primary Care Patient Safety Translational Research Centre<br>Howard, Rachel; Esplanade Surgery<br>Avery, Anthony; University of Nottingham, School of Medicine<br>Rodgers, Sarah; University of Nottingham, Division of Primary Care<br>Ashcroft, Darren; University of Manchester, Centre for Pharmacoepidemiology and Drug Safety, School of Health Sciences; University of Manchester, NIHR Greater Manchester Primary Care Patient Safety Translational Research Centre
**Primary Subject Heading**:	General practice / Family practice
Secondary Subject Heading:	Qualitative research, Sociology, Health services research
Keywords:	Prescibing, Information technology < BIOTECHNOLOGY & BIOINFORMATICS, PRIMARY CARE, Strong Structuration Theory, Medicines optimisation

SCHOLARONE™
Manuscripts

Understanding the implementation and adoption of an information technology intervention to support medicines optimisation in primary care: qualitative study using strong structuration theory

Corresponding author:-

Mark Jeffries , Centre for Pharmacoepidemiology and Drug Safety, School of Health Sciences, University of Manchester, Manchester, United Kingdom;

mark.jefferies@manchester.ac.uk

0161 275 3680

Denham L Phipps [1,2]

Rachel L Howard [3]

Anthony J Avery [4]

Sarah Rodgers [4]

Darren M Ashcroft [1,2]

[1]Centre for Pharmacoepidemiology and Drug Safety, School of Health Sciences, University of Manchester, Manchester, United Kingdom

[2]NIHR Greater Manchester Primary Care Patient Safety Translational Research Centre, University of Manchester, Manchester Academic Health Sciences Centre (MAHSC), United Kingdom

[3] Research Pharmacist, Isle of Wight, United Kingdom

[4] Division of Primary Care, University of Nottingham, Nottingham, United Kingdom

Word count: 4683

For peer review only - http://bmjopen.bmj.com/site/about/guidelines.xhtml

For peer review only

ABSTRACT

Objectives

Using strong structuration theory, we aimed to understand the adoption and implementation of an electronic clinical audit and feedback tool to support medicines optimisation for patients in primary care.

Design

Qualitative case study design. Transcripts were analysed using thematic analysis.

Setting

Clinical commissioning group in the South of England.

Participants

Four focus groups and six semi-structured interviews were conducted with eighteen participants purposively sampled from a range of stakeholder groups (general practitioners, pharmacists, patients and commissioners).

Results

Using the system could lead to improved medication safety but use was determined by broad institutional contexts, by the perceptions, dispositions and skills of users and by the structures embedded within the technology. These included: perceptions of the system as new and requiring technical competence and skill; the adoption of the system for information gathering; and interactions and relationships that involved individual, shared or collective use. The dynamics between these external, internal and technological structures affected the adoption and implementation of the system.

Conclusions

For peer review only

Successful implementation of information technology interventions for medicines optimisation will depend on a combination of the infrastructure within primary care, social structures embedded in the technology and the conventions, norms and dispositions of those utilising it. Future interventions, using electronic audit and feedback tools to improve medication safety, should consider the complexity of the social and organisational contexts and how internal and external structures can impact upon the use of the technology in order to support effective implementation.

For peer review only

Strengths and limitations of this study

- This is the first study to explore the implementation of electronic audit and feedback systems to improve medication safety in primary care using strong structuration theory.
- Strong structuration theory was found to be particularly valuable for unpicking why the system was used and the different motivations, ambitions, aims and attitudes of a range of stakeholders.
- Strong structuration theory also revealed the complex contextual background in which the system was implemented and how that implementation was informed by wider contexts.
- This work should inform policy makers, information technology system designers and those working in primary care about how to optimise the implementation of such systems in the future.

INTRODUCTION

Prescribing of medicines to patients is the most common clinical intervention in primary care. However, with high volumes of medicines prescribed in primary care [1], the prevalence of repeat prescribing and the increased burden and complexity of multimorbidity and related polypharmacy [2,3], there is an increased likelihood that prescribing or monitoring errors can occur [4,5]. Recent studies using prescribing safety indicators to investigate the prevalence of hazardous prescribing in primary care found 5.2 to 5.5% of patients to be at risk of potentially hazardous prescribing and 7.6

For peer review only - http://bmjopen.bmj.com/site/about/guidelines.xhtml

to 11.8% of patients not receiving recommended monitoring tests [6,7]. Some medication errors may not lead to harm; however, approximately 13% of patients have experienced an adverse drug event after receiving prescription medication in primary care, and many of those have been serious enough for patients to seek medical assistance at hospital [8,9]. The monitoring of patients in receipt of prescription medication is therefore considered important in order to avoid potentially serious adverse drug events.

Healthcare information technology (IT) systems may be useful for monitoring such medication usage. However, the implementation of such IT has not always been successful, with technology being resisted, not used effectively or used differently than was planned. Previous research has suggested that reasons for this might reside in the design and functionality of the technology. Poorly designed or implemented IT systems have been seen to create cognitive overload [10], and disrupt workflow [11]. Furthermore, IT systems may be utilised in ways unintended by developers in order to overcome problems with design. [12-14]. Such tailoring of systems suggests a dynamic where implementation involves interpretation and adaptation of systems to fit existing work practices, or changes to work practices in order to adapt to the new system [15-20].

This dynamic has been understood from a sociotechnical perspective, in which IT interventions are seen as being shaped by interactions between the technology, the users and social and organisational processes [21-23]. Such interactional views, rather than focusing only upon the functionality of systems, take into consideration the complex nature of healthcare and the cultural, social and organisational aspects of the workplaces [18, 24].

One way that sociotechnical theory has been developed and applied to the implementation of health care is through strong structuration theory [24]. Strong structuration theory understands the implementation of technology in terms of the ways it will be used or not and how actors draw upon particular resources to do so [25]. From a strong structuration viewpoint the implementation of healthcare IT is dependent upon a dynamic interplay of external structures, internal structures and the technology. External structures are built through social positions, practices and networks of social

For peer review only - http://bmjopen.bmj.com/site/about/guidelines.xhtml

For peer review only

relations. They could include local and national infrastructures such as national guidelines, political, economic and institutional contexts, professional codes of practice, as well as local work practices and interactions among stakeholders [25, 26]. Internal structures are manifest in two ways: firstly, as the skills, dispositions, ambitions, values and past experiences of actors; and secondly as the actors' knowledge of rules, conventions, obligations and social norms, which may involve partial understandings and past experiences, that inform how one is supposed to act [25-27]. Technology is seen as an equal actor within this, since technology has social structures built into it through procedures, material properties and standards that can enable or constrain use [25, 26, 28]. Technology can be therefore seen as shaping human actions by making certain actions possible [29].

This study examines the implementation of a new electronic medicines optimisation system (EMOS) in primary care that allows different stakeholders - general practitioners, Clinical Commissioning Group (CCG) managers, pharmacists, general practice managers, and patients - access to real time anonymized patient data including medical diagnoses, prescribed medications and laboratory test results. This facilitates electronic clinical audits to identify patients who are at risk of a medication-related adverse event, such as those who are on inappropriate combinations of drugs or who have not received appropriate monitoring. Within a health locality, the EMOS also allows clinicians and managers to audit prescribing practices across general practices and make comparisons against national guidelines. Patients have access to the system through a patient passport which allows them to view their medications and test results. Strong structuration theory has been previously used to understand the ways a large scale healthcare IT intervention, designed to assist patients and GPs to book hospital outpatient appointments, was resisted or adopted [28]. Using strong structuration theory we aimed to understand the implementation and adoption of the EMOS in primary care settings.

METHODS

Study design and setting

We used a case study design. The study case was a Clinical Commissioning Group (CCG) in the South of England, which was chosen because it was an early adopter of the EMOS and had all general

For peer review only - http://bmjopen.bmj.com/site/about/guidelines.xhtml

practices signed up to the system. In the English National Health Service (NHS), a Clinical Commissioning Group (CCG) is a clinically-led statutory NHS body responsible for the planning and commissioning of health care services for their local area, and cover groups of general practices within a local area. The sampling frame was people within the CCG's geographical area who represented the stakeholder groups. This included doctors, pharmacists, general practice managers and patients.

Recruitment and data collection

Individual participants were recruited on a purposive basis via the CCG or through community pharmacy networks, to represent the different stakeholder groups (see Table 1).

Table 1 – Case study participants

**Participants**	**Role**	**Use of EMOS**
**Interviews**		
GP1-INT	General Practitioner	In general practice and as prescribing lead of Clinical Commissioning Group (CCG) medicines management team
GP2	General Practitioner	In general practice and as respiratory lead at CCG
GP3	General Practitioner	In general practice
CCGP1 (additional observation as part of interview)	CCG Pharmacist	Medication reviews in care homes
CCGP2	CCG Pharmacist	CCG medicines management team
**Focus group A - General Practitioners**		
GP4	General Practitioner	In general practice
GP1-FG	General Practitioner	In practice and as prescribing lead of CCG medicines management team
**Focus group B – Community Pharmacists**		
CP1	Community Pharmacist	Aware of, but no access
CP2	Community Pharmacist	Aware of, but no access
CP3	Community Pharmacist	Aware of, but no access
CP4	Community Pharmacist	Aware of, but no access
**Focus Group C – Patients**		
Pt1	Patient	Access through patient passport
Pt2	Patient	Access through patient passport
Pt3	Patient	Access through patient passport

For peer review only - http://bmjopen.bmj.com/site/about/guidelines.xhtml

Pt4	Patient	Access through patient passport
**Focus Group D - General practice managers**		
GPM1	General Practice Manager	In general practice
GPM2	General Practice Manager	In general practice
GPM3	General Practice Manager	In general practice
GPM4	General Practice Manager	In general practice
**Declined to participate**. A number of possible participants were approached but declined to participate. Predominantly this was for reasons of time, workload or lack of use of the system. These included 2 pharmacist technicians, 2 GPs, 2 community pharmacists and 8 general practice managers.		

Potential participants were contacted by telephone or email. Five semi-structured interviews (lasting between 20-50 minutes) were conducted with three GPs and two CCG pharmacists, who were known to be using the system, between August and December 2014. Four homogeneous focus groups (lasting between 57-112 minutes) were conducted between September and December 2014, each with a specific group of stakeholders: GPs (2); community pharmacists (4); patients (4); and general practice managers (4). No repeat interviews were conducted. In the interviews and focus groups we explored experiences of working with the EMOS, perceptions of the system, benefits and drawbacks, the organisational structures and roles required for its use and the circumstances under which it was considered most effective. Data collection continued until saturation was reached and no new themes emerged from the interviews and focus groups. The interviews and focus groups were carried out by a male research associate in medication safetytrained and experienced in qualitative health research who holds an MSc in Health Psychology (MJ). The focus groups were co-facilitated by a female freelance research pharmacist experienced in qualitative methodology and who holds a PhD in Medicines Safety in Primary Care (RLH). The researchers were not known to the participants prior to the study. Four interviews were conducted by telephone and one at the CCG offices. The focus groups were conducted at the CCG offices or at a local hotel solely with the participants, RLH and MJ present. All participants gave written informed consent to take part in the study, and for the interviews and focus groups to be audio recorded and transcribed verbatim. Ethical approval for the study was granted by the Preston NHS Research Ethics Committee (reference 14/NW/0113).

For peer review only - http://bmjopen.bmj.com/site/about/guidelines.xhtml

Analysis

The analysis was thematic using a template analysis approach [30]. An a priori set of thematic codes based upon strong structuration theory was developed from the literature [25, 26, 28, 31]. These included: external structures such as national or local infrastructures; interactions, including relationships, conflicts and communication; the internal structures of agents including dispositions, skills, attitudes and cognitive demands; rules and contextuality including routines, social norms and regulations and technological structures including the social structures built into the technology. This set of codes was applied to the transcripts using the QSR NVivo 10 application. The coded extracts were then analysed and emerging themes highlighted which formed the basis of the results detailed below.

RESULTS

The adoption and implementation of the EMOS was dependent upon a dynamic mix of external structures, internal structures and the material properties embedded in the technology (as illustrated in Figure 1). External infrastructures, the motivations of users and the material properties of the EMOS facilitated information gathering. Perceiving the system as new could lead to resistance and the maintenance of habitual behaviours. Use was dependent upon interactions and relationships between users. Use could be further constrained by conceptualising the system as requiring technical competence.

INSERT FIGURE 1

The ways in which the EMOS was implemented and adopted were conceptualised in four broad thematic categories: Adoption of the system for information gathering; perceptions of the system as new; perceptions of the EMOS as requiring technical competence; and the interactions and relationships that involved individual or collective use of the technology.

Adoption of the system for information gathering

For peer review only - http://bmjopen.bmj.com/site/about/guidelines.xhtml

The EMOS facilitated the efficient acquisition of information relating to the appropriateness of prescribing for individual patients. External structures provided the conditions for the use of the technology; specifically through the requirements of national policies relating to safe medicines use as set down by national infrastructures and the CCG's responses to those requirements. The CCG was motivated to carry out audits of prescribing, and much of the data extracted through such audits were used to benchmark the CCG against these national policies and targets. This auditing was in turn determined by the policy and institutional climate that required the reporting of such auditing, the setting of certain guidelines and targets, and the adherence to those. This further led to the CCG utilising the technology in a local context to monitor prescribing behaviour in practices in response to local initiatives. External structures such as national or local "initiatives" worked with the internal structures (in this instance the motivations of the CCG to report in response to these "initiatives") and the material properties of the technology, to more swiftly identify patients registered with general practices that met the relevant prescribing safety audit. The technology shaped the ability to do this; the material properties of the system allowing for extensive searches of electronic health records across multiple general practices in a relatively short space of time. According to the following extract from an interview with a CCG pharmacist the technological structures enabled the collection of data in a more efficient and timely fashion.

> *" [...]it's a way of being able to gather pseudo-anonymised individual patient data and relate it to ideas and thoughts around initiatives that CCG or the medicines management team are looking at that perhaps has been identified or highlighted nationally, or locally and it can all be done relatively quickly within a few seconds if necessary. So you don't have to trawl round 17 different practices (CCGP2)*

Centrally, in a form of pay for performance initiative, the CCG made the EMOS part of a *"GP incentive scheme to engage with alerts in a meaningful way"* (GP1-INT) and this was conceptualised as *"trying to sort of get some more traction"* (GP1-INT). Guidelines and documents concerning strategies for prescribing framed the possibilities for use "*to actually monitor the progress against a sort of target outcome"* (GP1-INT). The functionality within the EMOS allowed for the benchmarking

For peer review only - http://bmjopen.bmj.com/site/about/guidelines.xhtml

across the CCG. This in turn provided for structures that could be utilised by the CCG to encourage practices to use the system, and an infrastructure that supported their own activities in monitoring prescribing behaviour and to *"reward good prescribing"* (GP1-INT).

> *"if there are some practices that are demonstrating very good prescribing, then we've picked those out as well and highlighted those to act as a kind of beacon of hope for everybody else". (CCGP2)*

The system also allowed for communication channels and feedback where contact with practices would be made through the system or as a result of alerts being sent out by email. Such communication, between the clinicians placed centrally at the CCG and the individual GP practices, enabled the CCG to monitor prescribing as *"a way of looking at the map"* (GP2) as well as the use of the system by *"tracking our advice in those practices"* (GP1-INT). The codes and material properties of the system facilitated monitoring in that logging on to the system indicated engagement with it. This in turn allowed the CCG to further monitor and audit prescribing patterns since they could swiftly see which practices had responded to alerts and *"[could] have some kind of objective measure that [gave them] some idea as to who's perhaps even more engaged than others"* (CCGP2). The ambitions and motivations of the CCG to monitor prescribing acted as an internal structure to work *"very hard to get the uptake of that better"* (CCGP2) and in "*trying to persuade our clinicians to use it so that we get a much more real time feedback."* (CCGP2). Furthermore this combination of technological infrastructure and the ambitions of the CCG created a new internal structure in the form of a convention for using the system.

> *"(When) the GP logs onto the Eclipse system and there's a little tick box to say patient reviewed [...]. Now some practices are doing that as a regular routine exercise, so that means that tracking our advice in those practices is very easy and what it does allow you to do as well is not to send the same alert out to the same practice again" (GP1-INT).*

Perceptions of the system as new

For peer review only - http://bmjopen.bmj.com/site/about/guidelines.xhtml

Using the EMOS was characterised as a new practice that would require new approaches. Resistance towards the system was thus justified by characterising existing behaviours as ingrained. Here habits and ways of doing things that were presented by one GP as "*the old fashioned way"*(CCGP2), provided for a limited use of the system. One such disposition was around their prescribing habits which they described as "*conservative"* (CCGP2). This allowed for a limited use of the EMOS, in which most alerts would not require action because prescribing behaviour was already "*protective of patients*"(CCGP2). Similarly, as the following extract illustrates, non-use of the system resulted from habitual accustomed practice of using other systems, pre-existing routines and repetitive ways of doing things

> *I think the trouble is Eclipse is another thing you have to log into along with the other 20 things you log into every day, and you're so used to using your other clinical system all the time."(GPM3)*

The CCG pharmacists were concerned that GPs would otherwise avoid using the system. It was assumed that GPs, in addition to, training on the system, needed persuasion in order to "*just [get] them to use it as habit"* (CCGP1).

> *"but we have had a situation where the GP said, oh, I'm not sure if I'll have time to look on Eclipse, but you can't spoon feed them everything"(CCGP1)*

Structures could shape the ways things were done. Workplace routines and practices, such as the prioritisation of work schedules, acted as constraints or enablers to the use of the new system. Here this GP highlighted contingencies within the structures associated with the *"special circumstances of my workplace"* (GP4) which allowed for a range of actions from side-lining the alert through to reviewing the patient. In this way the duality of structure - the specific demands of his work - and his agency - his interaction with the alerts in the EMOS - both governed his act of utilising the system and the extent and character of that utilisation.

For peer review only - http://bmjopen.bmj.com/site/about/guidelines.xhtml

> *"[...] it can depend on the nature of the alerts, how urgent it seems, and the special circumstances of my workplace[...] some things might actually get side-lined for a few weeks if they're not clinically urgent, but [...]the next time I catch up with my paperwork then I'll dig up that alert[...]and review the situation (GP4)*

For the CCG pharmacist, undertaking medication reviews in care homes the system changed the way they worked because *"if necessary if there's something that comes up on Eclipse whilst we're there we can, rather than having to go back to the surgery first, check it and then make a decision"* (CCGP1). In this way the technology shaped their actions. Furthermore the technological structures in the EMOS and the internal structures led to new shared decision making, use and outcome.

> *"We can look on Eclipse and most of the time it's on Eclipse and we can answer the question there and then. For example, we had a patient who was on Memantine, who was a really not very well gentleman,[...] so we phoned the GP straightaway."(CCGP1)*

Perceptions of the EMOS as requiring technical competence

The EMOS was conceptualised as a "clever" system that could conduct complex searches, but would require technical knowledge on the part of users in order to do so. This allowed for this GP's limited use of the system when combined with an understanding of his own abilities to use the system;

> *"That's how I become accustomed to doing things, which is perhaps why I then don't use Eclipse, because I do think I might not have the ability and the power of making the use of a more powerful tool. But, perhaps I have also then learned useful habits with the old fashioned way." (GP4)*

Non-use of the system was associated with the cognitive and physical demands associated with using the EMOS and finding time to learn how to get the best out of it. This further conceptualised the system as complex requiring time, training and *"proper teaching"* (GPM2) to gain the expertise required to utilise it.

For peer review only - http://bmjopen.bmj.com/site/about/guidelines.xhtml

"And if you had the time to log into it and go oh, what does this do? What does that do? […] You train your audit clerk who runs all sorts of searches and does all sorts of audit work, you could have the time to show her and teach her,[…] I'd love to have the time to tinker with,(the system).[…] You'd need time to play with it and time to…proper teaching, proper (training) showing us what it does" (GPM2)

The conceptualisation of the EMOS as requiring technical competence was related to structures embedded within the technology that allowed for or constrained its use. This could either empower users and thus facilitate further use or could undermine that agency.

"And also I'm computer literate and I can work out, I can problem solve because I'm reasonably well educated, if you were talking about average population here, they would either give up, they would probably have given up when they couldn't log in" (Pt2)

This service user conceptualises the system as difficult and one that required her abilities as a "*computer literate*" to use it. This required an interaction of her capabilities and the structures within the system to engage with it and difficulties with logging in was perceived to be a potential constraint for other users.

Interactions and relationships: Individual, shared and collective use of the technology

There were variations in the ways the technology was used within collaborative networks of social relations. Different general practice staff took responsibility for using the technology; use depended upon shared or collective roles, or upon a hierarchical allocation of access.

For service users, using the technology was determined by networks of social relations. This was expressed as having support from medical professionals to understand the system.

"But I think the important thing is before you sort of almost start using it, you do need that kind of intervention from a medical practitioner in some way to actually help you with the things you need to know" (Pt1)

For peer review only - http://bmjopen.bmj.com/site/about/guidelines.xhtml

Within general practices there was variation in who took responsibility for the EMOS. On receiving an alert through the system one practice manager would then *"pass it on to the GP and get them to respond to me" (GPM 2)* and that *"the doctors don't access it at all.[…] I'm the only one that, yeah, has anything to do with it."(GPM 2)* Another remarked:

> *"I get the alert the same way through the email, I identify the patient [...] then mine goes to the GP. But the GP actions it, I don't have any more responsibility for it after that […] They go into Eclipse, they do it, […] my job is just to literally give them the information and they do the rest." (GPM1)*

Such variation was driven by the conventions and norms associated with work practices. In different general practices individuals were assigned to different roles and responsibilities often based on what worked best for the practice.

> *"one of the GPs has been nominated within our practice to take that lead in the same way that we break our workload down in other areas; you be the lead for this and tell us if there's anything we all need to know and share the workload." (GPM4)*

The allocation of access to the EMOS limited its use. Community pharmacists did not have access to the system. Perceived social norms were seen as *"historically a barrier"* (CP2) that perpetuated that lack of access .Community pharmacists attributed this barrier to GPs seeing themselves as "*as the custodians of the patient record"* (CP2).

> *"I think there always has been a conflict because GPs often see themselves as the custodians of the patient record and even though the information in that patient record, even abbreviated information is incredibly useful for community pharmacists, they've never successfully managed to allow us access and this is going back to EPS [Electronic Prescription Service], this is what EPS promised and it's never happened." (CP2)*

For peer review only - http://bmjopen.bmj.com/site/about/guidelines.xhtml

There was a perception that the system was a tool for the CCG. This differential access meant that the system had not been used in some general practices. There were however perceptions that the system had *"evolved"*.

> *"I think that's what it was [...] originally purchased…or the agreement with Eclipse was originally for the meds management team to use it as a tool for them [...] And I think Eclipse has evolved since that happened [...] And I don't think any of us have kept up with how Eclipse has evolved and what else it can now do."(GPM3)*

Such changes were related to social norms around ownership and conventions concerning how the system would be used; centrally by the CCG to look at prescribing patterns across practices, and by individual practices of their own prescribing audits. As the system evolved there were perceptions that it could do more. In this way, perceptions of the technological structures and material properties of the technology drove the ambitions of some users to learn more about the potential uses of the system which opened up access to different users.

DISCUSSION

Much of the previous literature on interventions to improve medication safety has focused upon secondary healthcare settings and electronic audit and feedback systems of the kind examined in this study are under-reserached in primary care. A particular strength of this study is the use of strong structuration theory which was found to be a useful theoretical approach to studying the implementation and adoption of the EMOS. In applying this theoretical approach we were able to see the differences in motivations, ambitions, aims and attitudes of different actors from different stakeholder groups towards the IT intervention. Strong structuration theory could also reveal the complex contextual background in which the EMOS was implemented and revealed how the implementation was informed by wider contexts. Hence we were able to understand that the successful adoption of the EMOS was not merely dependent upon agents but upon the complex terrain in which it was implemented. Previous studies using this approach have focused upon large national IT projects where institutional contexts might be considered to have more impact [26, 32]. We found however that in a smaller scale project, wider

For peer review only - http://bmjopen.bmj.com/site/about/guidelines.xhtml

policy institutional contexts did impact upon the implementation and adoption of the IT for example through the CCG's response to the requirements of national policies. In this way the use of the system depended on other factors alongside the dispositions of the users.

There are several limitations to this work which present further opportunities future research examining the adoption and implementation of electronic audit and feedback systems to improve medication safety in primary care settings. In understanding the contexts in which the EMOS was adopted it would have been useful to have looked at policy documentation. It has been suggested that studies such as these explore wider social contexts through analysis of background data and through ethnographic observation [29, 33].Though we conducted one observation with a CCG pharmacist this was only as an extension of the interview with that participant to elicit some further understanding of how they used the EMOS. A number of naturalistic observations would have been useful in unpicking contexts and agents' choices and actions in using the system.

Relationship with previous literature

Primary care settings are governed by institutional norms, measures, rules and traditions, habits and behaviours [24, 34]. Some of these are embedded in local rules and conventions associated with the different working dynamics of individual practices and others are found in regulations and governance associated with wider economic and institutional contexts [24, 35]. In the present study we found that norms and social conventions could limit the use of the EMOS. Previous literature has established the role of social, organisational and work practices in the adoption of IT, [11, 12] others have focused upon functionality of design and tailoring to users [13, 36] or upon top-down implementation [36 , 37]. Previous research has indicated that emphasis upon training might also construct end-users as the problem [38]. Sociotechnical approaches to the implementation of IT have offered insight to the importance of the organisational and social contexts in which technology is delivered [16]. A focus upon interoperability, work practices and system usability suggests that poor adoption of IT is related to users or the system [14, 36]. This misses how interactions and relationships between contexts, users and the technology might work and how the implementation of IT is a social practice [39]. In this study, the

For peer review only - http://bmjopen.bmj.com/site/about/guidelines.xhtml

networks of social relations impacted upon the use of the system. Much of the previous sociotechnical literature does not seek to explain how a dynamic blend of wider political contexts, local priorities, the dispositions of users and the available properties of the technology work together. For instance whilst highlighting the importance of work practices and how technology needs to be embedded into pre-existing routines, previous literature, with notable exceptions, [24, 28, 29] has not seen these dynamically linked to wider contexts particularly in the context of medication safety in primary care. In our study whilst national guidelines might necessitate the monitoring of prescribing, the EMOS would not have been used to undertake this without the material properties within it and the ambitions of individuals at the CCG.

Implications of the findings

Strong structuration theory would argue that individual agency is dependent upon knowledge of rules and conventions. As IT is implemented, new rules and conventions are established, adapted or rejected. In our study it was seen that communication and feedback through the EMOS provided the CCG with the ability to monitor practices and to gain knowledge of which practices were engaged in using the system to improve medication safety. In this way the use of the system created new internal structures concerning such social rules and conventions. Similarly, in previous literature, information systems have been associated with enabling managers to capture information, place local clinicians under surveillance and make their actions calculable [39]. Furthermore an effect of such surveillance is for individuals to adapt their own behaviour to ensure they act legitimately [40].

This study highlights how healthcare IT interventions are implemented and adopted in a complex social and organisational context. Interventions that are top down and perceived as tools of managerial control are less likely to be effective than those that take into consideration existing local practices and the ambitions and attitudes of those who will use the technology.

CONCLUSION

For peer review only - http://bmjopen.bmj.com/site/about/guidelines.xhtml

Our study examines the implementation and adoption of an IT system for medicines optimisation in primary care. It was found that the dynamic combination of external, internal and technological structures impacted upon the adoption and implementation of the system. Information technology interventions for medicines optimisation should consider how utilisation may depend on a combination of the infrastructure within primary care, social structures embedded in the technology and the conventions, norms and dispositions of those utilising it.

Figures

Figure 1. Strong structuration theory and the use of the EMOS

Figure legend: Interaction between internal, external and technological structures that determined the use of the EMOS

Abbreviations

IT: Information technology; GP: General practitioner; EMOS: Electronic medicines optimisation system; CCG: Clinical commissioning group.

Acknowledgments

We are grateful to all participants who kindly gave their time.

Contributors

All authors were involved in the design of this study. MJ led on recruitment of participants, data collection, analysis of the data and drafting of the article. RLH helped co-facilitated focus groups. DLP, RLH. SR, AJA and DMA also made contributions to analysis and interpretation. All authors revised the article critically and approved the final version to be published.

Funding

For peer review only - http://bmjopen.bmj.com/site/about/guidelines.xhtml

This research was funded by the National Institute for Health Research (http://www.nihr.ac.uk) through the Greater Manchester Primary Care Patient Safety Translational Research Centre (NIHR GM PSTRC), grant number gmpstrc-2012-1.

Disclaimer

The views expressed are those of the author(s) and not necessarily those of the NHS, the NIHR or the Department of Health**.**

Competing interests

The authors declare they have no competing interests.

Ethical approval and consent to participate

All participants gave informed written consent to take part in the study, and for the interviews and focus groups to be audio recorded and transcribed verbatim . Ethical approval for the study was granted by the NHS National Research Ethics Service (reference 14/NW/0113)

Availability of data

Data cannot be shared because participants did not consent to this. In addition since this is a small case study, involving small numbers of participants, there is a possibility that material in the transcripts could identify participants.

For peer review only

References

1.HSCIC. Prescriptions Dispensed in the Community: England 2004-2014. London: Health and Social Care Information Centre; 2015.

For peer review only - http://bmjopen.bmj.com/site/about/guidelines.xhtml

2. Naylor C, Imison C, Addicott C, et al. Transforming our health care system: Ten priorities for commissioners. London: The King's Fund; 2015

3. Guthrie B, Makubate B, Hernadez-Santiago V, et al. The rising tide of polypharmacy and drug-drug interactions: population database analysis. 1995-2010 *BMC Med.* 2015; 13:74.

4. Avery AJ, Ghaleb, M, Barber N et al. The prevalence and nature of prescribing and monitoring errors in English general practice: a retrospective case note review. *Br J Gen Pract.* 2013; 63(613): e543-e553

5. Avery AJ, Sheikh A, Hurwitz B et al. Safer medicines management in primary care. *Br J Gen Pract.* 2002; 52 Suppl: S17-S22.

6. Akbarov A, Kontopantelis E, Sperrin M, et al. Primary care medication safety surveillance with integrated primary and secondary care electronic health records: a cross-sectional study. *Drug Saf.* 2015; 38:7: 671-682

7. Stocks SJ, Kontopantelis E, Akbarov A, et al. Examining variations in prescribing safety in UK general practice: cross sectional study using the Clinical Practice Research Datalink. *BMJ* 2015;351:h5501

8. Taché VS, Sönnichsen A, Ashcroft DM. Prevalence of adverse drug events in ambulatory care: a systematic review. *Ann Pharmacother*. 2011; 45; 977-989.

9. Kongkaew C, Hann M, Mandal J, et al. Risk factors for hospital admissions associated with adverse drug events. *Pharmacotherapy*. 2013; 33(8); 827-37.

10. Zahabi M., Kaber, DB, Swangnetr M. Usability and Safety in Electronic Medical Records Interface Design: A Review of Recent Literature and Guideline Formulation. *Hum Factors*. 2015; 57(5); 805-34.

For peer review only - http://bmjopen.bmj.com/site/about/guidelines.xhtml

11. Hayward J, Thomson F, Milne H, et al. "Too much, too late": mixed methods multi-channel video recording study of computerized decision support systems and GP prescribing. *J Am Med Inform Assoc*. 2013; e76-e84

12. Burgin A, O'Rourke R, Tully MP. Learning to work with electronic patient records and prescription charts: experiences and perceptions of hospital pharmacists. *Res Soc Adm Pharm.* 2014; 10(5); 741-55.

13. Harvey J, Avery AJ, Hibberd R, et al. Meeting user needs in national healthcare systems: lessons from early adopter community pharmacists using the electronic prescriptions service. *BMC Med Inform Decis Mak.* 2014; 14,16

14.Garfield S, Hibberd R, Barber N. English community pharmacists' experiences of using electronic transmission of prescriptions: a qualitative study. *BMC Health Serv Res.* 2013; 13; 343

15. Swinglehurst D, Greenhalgh T, Russell J,et al. Receptionist input to quality and safety in repeat prescribing in UK general practice: ethnographic case study. *BMJ* 2011;343:d6788.

16. Peiris D, Usherwood T, Weeramanthri T, et al. New tools for an old trade: a socio-technical appraisal of how electronic decision support is used by primary care practitioners. *Socio Health Illn.* 2011; 33(7): 1002-1018

17. Timmermans S, Berg M. The practice of medical technology. *Socio Health Illn.* 2003; 25:97-114

18. Clegg C. Sociotechnical principles for system design. *Appl Ergon*. 2000; 31: 463-477

19. Berg M. Problems and promises of the protocol. *Soc Sci Med.*1997; 44(8):1081-8

20. Clegg C. Shepherd C. "The biggest computer programme in the world... ever!": time for a change of mindset. *J Inform Technol*. 2007; 22: 212-221

For peer review only - http://bmjopen.bmj.com/site/about/guidelines.xhtml

21. Aarts A. Sociotechnical perspective of electronic prescribing in Tully MP, Dean Franklin B (Eds) Safety in Medication Use. Boca Raton FL: CRC Press 2015

22. Berg M. Implementing information systems in health care organizations: myths and challenges. *Int J med inform*. 2001; 64: 143-156

23. Oroviogoicoechea C, Watson R. A quantitative analysis of the impact of a computerised information system on nurses clinical practice using a realistic evaluation framework. *Int J med inform*. 2009; 78:839-849

24. Greenhalgh T, Swinglehurst D, Stones R. Rethinking resistance to big IT: a sociological study of why and when healthcare staff do not use nationally mandated information and communication technologies. *Health Serv Deliv Res*. 2014; 2(39)

25. Stones R. Structuration Theory. Basingstoke: Palgrave-Macmillan; 2005.

26. Greenhalgh T, Stones R. Theorising big IT programmes in healthcare: strong structuration theory meets actor–network theory. *Soc Sci Med* 2010;70:1285–94.

27. Hinder S, Greenhalgh T. "This does my head in" Ethnographic study of self-management by people with diabetes *BMC Health Serv Res* 2012; 1:83

28. Greenhalgh T, Stones R, Swinglehurst D. Choose and Book: a sociological analysis of 'resistance' to an expert system, *Soc Sci Med* 2014; 104:210-19

29.Greenhalgh T, Swinglehurst D. Studying technology uses as a social practice: the untapped potential of ethnography. *BMC Med*. 2011; 9:45

For peer review only

For peer review only - http://bmjopen.bmj.com/site/about/guidelines.xhtml

30. King, N. 'Doing template analysis', in G.Symon and C.Cassell (eds.) Qualitative Organizational Research: Core Methods and Current Challenges. London: Sage 2012.

31. Hardcastle MR, Usher KJ, Holmes CA, An overview of structuration theory and its usefulness in nursing research. *Nurs Philos* 2005; 6: 223-234

32. Cresswell KM, Bates DW, Sheikh A. Ten key considerations for the successful implementation and adoption of large-scale health information technology. *J Am Med Inform Assoc.* 2013; 0, 1-5

33. Swinglehurst D, Greenhalgh T, Myall M, et al. Ethnographic study of ICT-supported collaborative work routines in general practice. *BMC Health Serv Res*. 2010;10:348

34. Daker-White G, Hays R, McSharry J, et al. Blame the patient, blame the doctor? A meta-synthesis in qualitative studies of patient safety in primary care. *PLOS One*. 2015; 10(8), e0128329

35. Winthereik BR, van der Ploeg I, Berg M. The electronic patient record as a meaningful audit tool: accountability and autonomy in general practitioner work. *Sci Technol Hum Values* 2007;32:6–25.

36. Cresswell KM, Worth A, Sheikh A. Integration of a nationally procured electronic health record system into user work practices *BMC Med Inform Decis Mak*. 2012;12:15

37. Sheikh A, Cornford T, Barber N, et al. Implementation and adoption of nationwide electronic health records in secondary care in England: final qualitative results from prospective national evaluation in "early adopter" hospitals. *BMJ*. 2011; 343:d6054

38. Waterson P. Health information technology and sociotechnical systems: A progress report on recent developments within the UK National Health Service (NHS). *Appl Ergon*. 2014; 45: 150-161

39. Greenhalgh T, Russell J. Why do evaluations of eHealth programs fail? An alternative set of guiding principles. *PLOS Med*. 2010: 7:e1000360

For peer review only - http://bmjopen.bmj.com/site/about/guidelines.xhtml

40 . Doolin B. Power and resistance in the implementation of a medical management information system. *Inform Syst J* 2004;14:343–62.

For peer review only

For peer review only - http://bmjopen.bmj.com/site/about/guidelines.xhtml

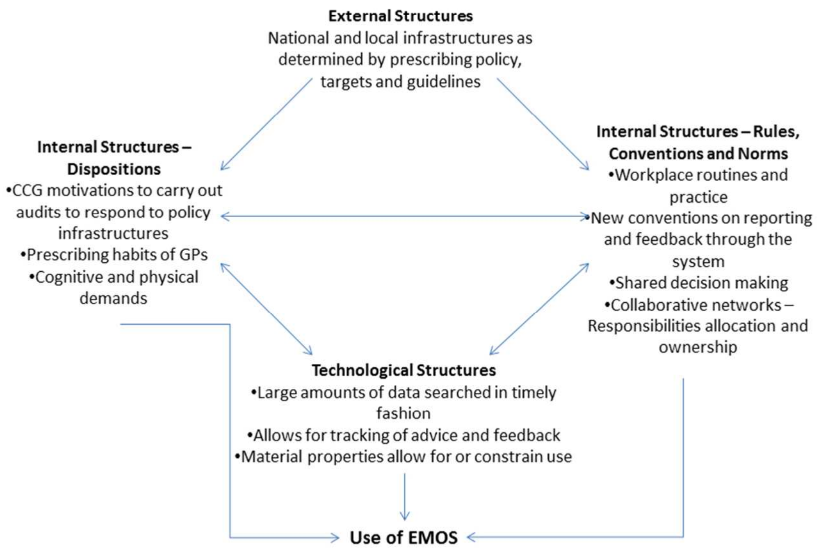

Strong structuration theory and the use of the EMOS: Interaction between internal, external and technological structures that determined use of the EMOS

275x190mm (96 x 96 DPI)

For peer review only

Understanding the implementation and adoption of an information technology intervention to support medicines optimisation in primary care: qualitative study using strong structuration theory

COREQ checklist

Note: in order to minimize the length of the manuscript, some of the details on the checklist (marked '*') are not included in the manuscript.

For peer review only

Guide question		*Response*	*Page number in manuscript*
1	Interviewer/facilitator	MJ conducted the interviews. MJ and RLH facilitated the focus groups	7
2	Credentials	MJ holds an MSc in Health Psychology<br>RLH holds a PhD in Medic8ines safety in Primary care	7
3	Occupation	MJ : Research Associate in medication safety;<br>RLH: freelance research pharmacist	7
4	Gender	MJ male; RLH female	7
5	Experience and training	Both researchers have previous experience of undertaking qualitative research in healthcare at PhD and postdoctoral level	7
6	Relationship established	The researchers were not known to the participants prior to the study	7
7	Participant knowledge of the researcher	Participants were made aware of the reasons for doing the research via the information which was sent to the participant prior to the interview	*
8	Researcher characteristics	The researchers had identified the study topic as part of larger programmes of work in their research groups, medication safety in primary care.	7
9	Methodological orientation and theory	Strong structuration theory. The analysis was thematic using template analysis.	4-5, 7-8
10	Sampling	Individual participants were recruited on a purposive basis via the study CCG or through community pharmacy networks. All participants were chosen to fit the sampling frame (people within the CCG's geographical area who represented the stakeholder groups: pharmacists, doctors, general practice managers and patients)	6
11	Method of approach	Participants were approached by telephone or email	7
12	Sample size	19 participants	6-7
13	Non-participation	A number of possible participants were approached but declined to participate. Predominantly this was for reasons of time, workload or lack of use of the system. These included 2 pharmacist technicians, 2 GPs, 2 community pharmacists and 8 general practice managers.	7
14	Setting of data collection	Four interviews were conducted by telephone and one at the CCG offices, the focus groups were conducted at the CCG offices or at a local hotel.	7
15	Presence of non-participants	No non-participants were present	7

16	Description of sample	See Table 1 of the main manuscript	6-7
17	Interview guide	In the interviews and focus groups we explored experiences of working with the EMOS, perceptions of the system, benefits and drawbacks, the organisational structures and roles required for its use and the circumstances under which it was considered most effective. No pilot testing was undertaken due to the small scale nature of the study, the timescale of the study and the difficulties of recruitment.	7
18	Repeat interviews	None	7
19	Audio/visual recording	Audio recording only, with consent from the participant	7
20	Field notes	None	*
21	Duration	The interviews lasted between 20 and 50 mins. The focus groups lasted between 57-112 mins.	7
22	Data saturation	Data collection continued until saturation was reached and no new themes emerged from the interviews and focus groups.	7
23	Transcripts returned	No transcripts were returned to participants	*
24	Number of data coders	MJ coded the data but regular discussions codes were held with all authors	18
25	Description of the coding tree	A coding tree description is not given but details on a priori codes is included	8
26	Derivation of themes	A priori thematic codes were applied to the data and new themes emerged from the data. This is described in the analysis section	8
27	Software	QSRNvivo 10 software was utilised to manage the data	8
28	Participant checking	No	*
29	Quotations presented	Please see the results section of the manuscript	8-14
30	Data and findings consistent		8-14
31	Clarity of major themes		8-14
32	Clarity of minor themes		8-14

For peer review only

Understanding the implementation and adoption of an information technology intervention to support medicines optimisation in primary care: qualitative study using strong structuration theory

Journal:	*BMJ Open*
Manuscript ID	bmjopen-2016-014810.R1
Article Type:	Research
Date Submitted by the Author:	31-Jan-2017
Complete List of Authors:	Jeffries, Mark; University of Manchester, Centre for Pharmacoepidemiology and Drug Safety,School of Health Sciences; University of Manchester, NIHR Greater Manchester Primary Care Patient Safety Translational Research Centre<br>Phipps, Denham; University of Manchester, Centre for Pharmacoepidemiology and Drug Safety, School of Health Sciences; University of Manchester, NIHR Greater Manchester Primary Care Patient Safety Translational Research Centre<br>Howard, Rachel; Esplanade Surgery<br>Avery, Anthony; University of Nottingham, School of Medicine<br>Rodgers, Sarah; University of Nottingham, Division of Primary Care<br>Ashcroft, Darren; University of Manchester, Centre for Pharmacoepidemiology and Drug Safety, School of Health Sciences; University of Manchester, NIHR Greater Manchester Primary Care Patient Safety Translational Research Centre
**Primary Subject Heading**:	General practice / Family practice
Secondary Subject Heading:	Qualitative research, Sociology, Health services research
Keywords:	Prescibing, Information technology < BIOTECHNOLOGY & BIOINFORMATICS, PRIMARY CARE, Strong Structuration Theory, Medicines optimisation

Understanding the implementation and adoption of an information technology intervention to support medicines optimisation in primary care: qualitative study using strong structuration theory.

Corresponding author:-

Mark Jeffries, Centre for Pharmacoepidemiology and Drug Safety, School of Health Sciences, Faculty of Biology, Medicine and Health, University of Manchester, Manchester, United Kingdom;

mark.jefferies@manchester.ac.uk

0161 275 3680

Denham L Phipps [1,2]

Rachel L Howard [3]

Anthony J Avery [4]

Sarah Rodgers [4]

Darren M Ashcroft [1,2]

[1]Centre for Pharmacoepidemiology and Drug Safety, School of Health Sciences, University of Manchester, Manchester, United Kingdom

[2]NIHR Greater Manchester Primary Care Patient Safety Translational Research Centre, University of Manchester, Manchester Academic Health Sciences Centre (MAHSC), United Kingdom

[3] Research Pharmacist, Isle of Wight, United Kingdom

[4] Division of Primary Care, University of Nottingham, Nottingham, United Kingdom

Word count: 5681

For peer review only - http://bmjopen.bmj.com/site/about/guidelines.xhtml

ABSTRACT

Objectives

Using strong structuration theory, we aimed to understand the adoption and implementation of an electronic clinical audit and feedback tool to support medicines optimisation for patients in primary care.

Design

Qualitative study. Transcripts were analysed using template analysis.

Setting

Clinical commissioning group in the South of England.

Participants

Four focus groups and five semi-structured interviews were conducted with eighteen participants purposively sampled from a range of stakeholder groups (general practitioners, pharmacists, patients and commissioners).

Results

Using the system could lead to improved medication safety but use was determined by broad institutional contexts, by the perceptions, dispositions and skills of users and by the structures embedded within the technology. These included: perceptions of the system as new and requiring technical competence and skill; the adoption of the system for information gathering; and interactions and relationships that involved individual, shared or collective use. The dynamics between these external, internal and technological structures affected the adoption and implementation of the system.

Conclusions

For peer review only - http://bmjopen.bmj.com/site/about/guidelines.xhtml

Successful implementation of information technology interventions for medicines optimisation will depend on a combination of the infrastructure within primary care, social structures embedded in the technology and the conventions, norms and dispositions of those utilising it. Future interventions, using electronic audit and feedback tools to improve medication safety, should consider the complexity of the social and organisational contexts and how internal and external structures can impact upon the use of the technology in order to support effective implementation.

Strengths and limitations of this study

- This is the first study to explore the implementation of electronic audit and feedback systems to improve medication safety in primary care using strong structuration theory.
- Strong structuration theory was found to be particularly valuable for unpicking why the system was used and the different motivations, ambitions, aims and attitudes of a range of stakeholders.
- This was an exploratory study that relied mainly upon interview and focus group data from a number of key stakeholders located in one clinical commissioning group in England.
- Additional insights may have been gained by undertaking ethnographic observation to discover exactly the ways people utilised the Electronic Medicines Optimisation System (EMOS).

INTRODUCTION

Prescribing of medicines to patients is the most common clinical intervention in primary care. However, with high volumes of medicines prescribed in primary care [1], the prevalence of repeat prescribing and the increased burden and complexity of multimorbidity and related polypharmacy [2,3], there is an increased likelihood that prescribing or monitoring errors can occur [4,5]. Recent studies using prescribing safety indicators to investigate the prevalence of hazardous prescribing in primary care found 5.2 to 5.5% of patients to be at risk of potentially hazardous prescribing and 7.6

For peer review only - http://bmjopen.bmj.com/site/about/guidelines.xhtml

to 11.8% of patients not receiving recommended monitoring tests [6,7]. Some medication errors may not lead to harm; however, approximately 13% of patients have experienced an adverse drug event after receiving prescription medication in primary care, and many of those have been serious enough for patients to seek medical assistance at hospital [8, 9]. The monitoring of patients in receipt of prescription medication is therefore considered important in order to avoid potentially serious adverse drug events.

In the UK, national and local policies have set out recommendations for medication safety improvement [10-12]. "An Organisation with a Memory" [13] set out the necessity for the establishment of a patient safety culture within healthcare organisations. This emphasised the importance of organisational practices. Policy has also set out how the utilisation of information technology presents opportunities to fulfil medication safety requirements and that the contribution of information systems should be maximised [11]. This was further enhanced by Department of Health recommendations in the report “Building a Safer NHS for patients: Improving Medication Safety” [10], which recommended that steps to safer prescribing may include the implementation of effective IT systems particularly those systems that might highlight and give warnings to medical staff of prescription errors. Similar recommendations have suggested there is a need to develop systems that optimise the use of medicines and that this might include improved electronic decision support for clinicians [12]. Locally, the Clinical Commissioning Group (CCG) that formed the setting for this study operated a prescribing incentive scheme designed to improve the quality of prescribing, respond to the requirements of national guidelines, and reduce excessive prescribing and costs, which was incentivised by small financial rewards for general practices [14].

Healthcare information technology (IT) systems may be useful for monitoring medication usage. However, the implementation of such IT has not always been successful, with technology being resisted, not used effectively or used differently than was planned. Previous research has suggested that reasons for this might reside in the design and functionality of the technology. Poorly designed or implemented IT systems have been seen to create cognitive overload [15], and disrupt workflow [16]. Furthermore, IT systems may be utilised in ways unintended by developers, either to overcome

For peer review only - http://bmjopen.bmj.com/site/about/guidelines.xhtml

problems with design or as new uses for the technology become apparent [17-19]. However, such tailoring of systems suggests a dynamic where implementation actually involves interpretation and adaptation of systems to fit existing work practices, or changes to work practices in order to adapt to the new system [20-25]. In other words, the success or failure of an IT implementation could be seen as being shaped by interactions between the technology, the users and social and organisational processes [26-28].This sociotechnical view, rather than focusing only upon the functionality of systems, takes into account the complex nature of healthcare and the cultural, social and organisational aspects of the workplaces [22-29].

Strong structuration theory (SST) has been proposed as a way of examining these sociotechnical aspects of healthcare IT implementation [30]. It is based on Giddens' structuration theory, which proposed a relationship between structures (such as social norms, political and economic institutions) and agency (people's actions and choices) [31]. According to Stones [30], SST extends this structure - agency relationship to include the following elements (see figure 1):

- *External structures*, which are the physical social or economic context in which action is contemplated. External structures are built through social positions, practices and networks of social relationships [29, 32].These could include hierarchical relationships between employers and employees, professional roles, local and national guidelines, governance measures, regulations, professional codes of practice, as well as local work practices and interactions among groups of stakeholders [30, 32].
- *Internal structures*, which are manifest in two ways. Firstly, as the skills, dispositions, ambitions, attitudes, values, past experiences of actors and ways of viewing the world. Secondly, as the actors' knowledge of rules, conventions, obligations and social norms, which may involve partial understandings and past experiences. These inform how one is supposed to act in specific situations in the here and now, based upon the agents understanding of external structures [30, 32-33].
- *Agency,* which is how and why agents draw upon internal structures to act in particular ways in specific situations [32]

For peer review only - http://bmjopen.bmj.com/site/about/guidelines.xhtml

- *Outcome,* which is the way agency impacts on external or internal structures and how they are maintained or changed [32].

Stones and Greenhalgh [32] further explained the role of technology in SST: rather than there being symmetry between technology and human actors, they are instead separate and may act in different ways [32]. Technology incorporates procedures, codes, material properties and standards that can enable or constrain use [19, 32-34]; it is therefore seen as shaping human actions by making certain actions possible [35]. Previous studies [29, 34] suggest that SST can illuminate the implementation and adoption of information technology by understanding how people *"take action with respect to technologies"*; in other words, what people actually do with the systems and to what effect [32]. Strong structuration theory has been previously used to understand the ways a large scale healthcare IT intervention, designed to assist patients and General Practitioners (GPs) to book hospital outpatient appointments, was resisted or adopted [34].

INSERT FIGURE 1

This study uses SST to examine a new electronic medicines optimisation system (EMOS) [36] that was implemented in a primary care locality. The EMOS allows different stakeholders - general practitioners, Clinical Commissioning Group (CCG) managers, pharmacists, general practice managers, and patients - access to real time anonymized patient data including medical diagnoses, prescribed medications and laboratory test results. It comprises a secure patient database and a web-based user interface that extracts patient specific data from the general practice clinical record system. The interface provides a number of user functions; these include reviewing a specific patient health record, identifying patients who are at risk of a medication-related adverse event, such as those who are on inappropriate combinations of drugs or who have not received appropriate monitoring and carrying out clinical audits on a subset of patients [36]. The EMOS also allowed clinicians and managers in the health locality to audit prescribing practices across general practices and make comparisons against national guidelines. Patients have access to the system through a patient passport which allows them to view their medications and test results. In this context it was felt that SST would

For peer review only - http://bmjopen.bmj.com/site/about/guidelines.xhtml

unpick the ways in which users of the system drew upon their dispositions, attitudes skills and ambitions and upon their knowledge of and understanding of external structures to engage with the technology. Therefore, we aimed to examine the specific question: “in what ways did external, internal and technological structures impact upon the implementation and adoption of the EMOS?”

METHODS

Study design and setting

Our study used a qualitative design. The study setting was a CCG in the South of England, which was chosen because it was an early adopter of the EMOS and had all general practices signed up to the system. The CCG was relatively small in size (17 separate general practices, and approximately 140,000 patients). Medicines management activities at the CCG were undertaken by three clinical pharmacists (including participants CCGP1 and CCGP2) and two pharmacy technicians. Additionally one GP (participant GP1) operated as prescribing lead for the CCG. In the English National Health Service (NHS), a CCG is a clinically-led statutory NHS body responsible for the planning and commissioning of health care services for their local area, and cover groups of general practices within a local area. The sampling frame was people within the CCG’s geographical area who represented the stakeholder groups. This included doctors, pharmacists, general practice managers and patients.

Understanding the background

Prior to data collection we undertook actions to build a picture of the system and the context in which it was to be used. Authors MJ and RLH were given an overview of the system in a preliminary meeting with the study CCG prior to data collection. In addition MJ visited a separate CCG in the North of England that was utilising the EMOS. Web-based materials relating to the system were read prior to data collection [36].

Recruitment and data collection

For peer review only - http://bmjopen.bmj.com/site/about/guidelines.xhtml

Individual participants were recruited on a purposive basis via the CCG or through community pharmacy networks, to represent the different stakeholder groups (see Table 1).

Table 1 –Study participants

Participants	Role	How they used the EMOS
**Interviews**		
GP1-INT	General Practitioner	In general practice and prescribing lead for the Clinical Commissioning Group (CCG) .Worked with the medicines management team in supporting the adoption of the EMOS by the CCG. Used the EMOS to send alerts to GPs.
GP2	General Practitioner	In general practice and respiratory lead for the CCG. Utilised the EMOS to undertake audits of prescribing relating to respiratory conditions.
GP3	General Practitioner	In general practice
CCGP1 (additional observation as part of interview)	CCG Pharmacist	Utilised the EMOs to undertake medication reviews with care home patients
CCGP2	CCG Pharmacist	CCG medicines management team. Used the EMOS to run audits centrally at the CCG and then alert clinicians locally
**Focus group A - General Practitioners**		
GP4	General Practitioner	In general practice
GP1-FG	General Practitioner	In practice and as prescribing lead for the CCG
**Focus group B – Community Pharmacists**		
CP1	Community Pharmacist	Aware of, but no access
CP2	Community Pharmacist	Aware of, but no access
CP3	Community Pharmacist	Aware of, but no access
CP4	Community Pharmacist	Aware of, but no access
**Focus Group C – Patients**		
Pt1	Patient	Access through patient passport
Pt2	Patient	Access through patient passport
Pt3	Patient	Access through patient passport
Pt4	Patient	Access through patient passport
**Focus Group D - General practice managers**		
GPM1	General Practice Manager	In general practice
GPM2	General Practice Manager	In general practice
GPM3	General Practice Manager	In general practice
GPM4	General Practice Manager	In general practice

For peer review only

For peer review only - http://bmjopen.bmj.com/site/about/guidelines.xhtml

Potential participants were contacted by telephone or email. Five semi-structured interviews (lasting between 20-50 minutes) were conducted with three GPs and two CCG pharmacists, who were known to be using the system, and had specific roles that required the use of the EMOS between August and December 2014. Four homogeneous focus groups (lasting between 57-112 minutes) were also conducted between September and December 2014, each with a specific group of stakeholders: GPs (2); community pharmacists (4); patients (4); and general practice managers (4). No repeat interviews were conducted, although one GP was interviewed and also participated in a focus group. Each focus group was conducted with different a specific type of stakeholder, as this was felt to facilitate free and open discussion.

Topic guides for the interviews and focus groups were developed by reading relevant literature examining the implementation of information technology in healthcare settings [16, 17, 20, 23, 24.] Both interviews and focus groups were conducted to illicit individual thoughts and opinions and to promote discussion amongst specific homogenous groups of stakeholders. In the interviews and focus groups, we explored experiences of working with the EMOS, perceptions of the system, benefits and drawbacks, the organisational structures and roles required for its use and the circumstances under which it was considered most effective. Data collection continued until saturation was reached and no new themes emerged from the interviews and focus groups. The interviews and focus groups were carried out by a male research associate in medication safety trained and experienced in qualitative health research who holds an MSc in Health Psychology (MJ). The focus groups were co-facilitated by a female freelance research pharmacist experienced in qualitative methodology and with a PhD in Medicines Safety in Primary Care (RLH). The researchers were not known to the participants prior to the study. Four interviews were conducted by telephone and one at the CCG offices. The focus groups were conducted at the CCG offices or at a local hotel solely with the participants, RLH and MJ present. All participants gave written informed consent to take part in the study, and for the interviews and focus groups to be audio recorded and transcribed verbatim. Ethical approval for the study was granted by the Preston NHS Research Ethics Committee (reference 14/NW/0113).

For peer review only - http://bmjopen.bmj.com/site/about/guidelines.xhtml

Analysis

The analysis was thematic, using a template approach [37]. Template analysis involves the summarising of themes through a coding template. Often, template analysis begins with an *a priori* set of themes. New themes are then added, or existing themes revised, as data is iteratively analysed in a process of developing a template [37]. An *a priori* set of thematic codes based upon strong structuration theory was developed from the literature [30, 32, 34, 38.] These included: external structures such as national or local policies, guidelines and governance; interactions, including relationships, conflicts and communication; the internal structures of agents including dispositions, skills, attitudes and cognitive demands; rules and contextuality including routines, social norms and regulations and technological structures including the social structures built into the technology. This set of codes was applied to the transcripts by MJ and documented using the QSR NVivo 10 application. The coding template was then modified through successive readings and of the data and discussions with other authors. The template was then internally reviewed for completeness by MJ and DLP (who had independently reviewed all transcripts).

RESULTS

The ways in which the EMOS was implemented and adopted were conceptualised in four broad thematic categories: adoption of the system for information gathering; perceptions of the system as new; perceptions of the EMOS as requiring technical competence; and the interactions and relationships that involved individual or collective use of the technology.

Adoption of the system for information gathering

The EMOS facilitated the efficient acquisition of information relating to the appropriateness of prescribing for individual patients. External structures provided the conditions for the use of the technology; specifically through the requirements of national policies relating to safe medicines use as set down by national governance and guidelines and the CCG's responses to those requirements. The CCG was motivated to carry out audits of prescribing, and much of the data extracted through such

For peer review only - http://bmjopen.bmj.com/site/about/guidelines.xhtml

audits were used to benchmark the CCG against these national policies and targets. This auditing was in turn determined by the policy and institutional climate that required the reporting of such auditing, the setting of certain guidelines and targets, and the adherence to those. This further led to the CCG utilising the technology in a local context to monitor prescribing behaviour in practices in response to local initiatives. External structures such as national or local “initiatives” worked with the internal structures (in this specific instance the motivations of the CCG to report in response to these "initiatives") and the material properties of the technology, to more swiftly identify patients registered with general practices that met the relevant prescribing safety audit. The material properties of the system shaped the ability to conduct extensive searches of electronic health records across multiple general practices in a relatively short space of time. According to the following extract from an interview with a CCG pharmacist the technological structures enabled the collection of data in a more efficient and timely fashion.

> *"[...]it's a way of being able to gather pseudo-anonymised individual patient data and relate it to ideas and thoughts around initiatives that CCG or the medicines management team are looking at that perhaps has been identified or highlighted nationally, or locally and it can all be done relatively quickly within a few seconds if necessary. So you don't have to trawl round 17 different practices" (CCGP2)*

Centrally, in a form of pay for performance initiative, the CCG made the EMOS part of a *"GP incentive scheme to engage with alerts in a meaningful way"* (GP1-INT) and this was conceptualised as *"trying to sort of get some more traction"* (GP1-INT). Guidelines and documents concerning strategies for prescribing framed the possibilities for use "*to actually monitor the progress against a sort of target outcome"* (GP1-INT). The functionality within the EMOS allowed for benchmarking across the CCG. This in turn provided for structures that could be utilised by the CCG to encourage practices to use the system, and an infrastructure that supported their own activities in monitoring prescribing behaviour and to *"reward good prescribing"* (GP1-INT).

For peer review only - http://bmjopen.bmj.com/site/about/guidelines.xhtml

> *"if there are some practices that are demonstrating very good prescribing, then we've picked those out as well and highlighted those to act as a kind of beacon of hope for everybody else". (CCGP2)*

The system also allowed for communication channels and feedback, where contact with practices was made through the system or as a result of alerts being sent out by email. Such communication, between the clinicians placed centrally at the CCG and the individual GP practices, enabled the CCG to monitor prescribing as *"a way of looking at the map"* (GP2) as well as the use of the system by *"tracking our advice in those practices"* (GP1-INT). The codes and material properties of the system facilitated monitoring in that logging on to the system indicated engagement with it. This in turn allowed the CCG to further monitor and audit prescribing patterns since they could swiftly see which practices had responded to alerts and *"[could] have some kind of objective measure that [gave them] some idea as to who's perhaps even more engaged than others"* (CCGP2). The ambitions and motivations of the CCG to monitor prescribing acted as an internal structure to work *"very hard to get the uptake of that better"* (CCGP2) and in "*trying to persuade our clinicians to use it so that we get a much more real time feedback."* (CCGP2). Furthermore, this combination of technological infrastructure and the ambitions of the CCG created a new internal structure in the form of a convention for using the system.

> *"(When) the GP logs onto the Eclipse system and there's a little tick box to say patient reviewed [...]. Now some practices are doing that as a regular routine exercise, so that means that tracking our advice in those practices is very easy and what it does allow you to do as well is not to send the same alert out to the same practice again" (GP1-INT).*

In this way there were patterns of agent-technology relationships that reinforced a hierarchical agent - agent relationship within the network. The CCG managers were interacting with the technology to monitor prescribing since engagement at local clinician level with the system was encouraged by the CCG, because it provided further feedback to them, agent-technology relationships could build

For peer review only - http://bmjopen.bmj.com/site/about/guidelines.xhtml

through the system use as new agent -agent relationships between managers centrally at the CCG and local GPs.

Perceptions of the system as new

Using the EMOS was characterised as a new practice that would require new approaches. Resistance towards the system was thus justified by characterising existing behaviours as ingrained. Here, habits and ways of doing things that were presented by one GP as "*the old fashioned way*" (CCGP2) provided for a limited use of the system. One such disposition was around their prescribing habits, which they described as "*conservative*" (CCGP2). This allowed for a limited use of the EMOS, in which most alerts would not require action because prescribing behaviour was already "*protective of patients*"(CCGP2). Similarly, as the following extract illustrates, non-use of the system resulted from habitual accustomed practice of using other systems, pre-existing routines and repetitive ways of doing things.

> *"I think the trouble is Eclipse is another thing you have to log into along with the other 20 things you log into every day, and you're so used to using your other clinical system all the time."(GPM3)*

In a further example of agent-agent relationships associated with the use of the system, the CCG pharmacists were concerned that GPs would otherwise avoid using the system. It was assumed that GPs, in addition to training on the system, needed persuasion in order to "*just [get] them to use it as habit"* (CCGP1).

> *"but we have had a situation where the GP said, oh, I'm not sure if I'll have time to look on Eclipse, but you can't spoon feed them everything"(CCGP1)*

Social structures could shape the ways things were done. Workplace routines and practices, such as the prioritisation of work schedules, acted as constraints or enablers to the use of the new system. Here this GP highlighted contingencies within the structures associated with the *"special circumstances of my workplace"* (GP4) which allowed for a range of actions from side-lining the alert

For peer review only - http://bmjopen.bmj.com/site/about/guidelines.xhtml

through to reviewing the patient. In this way the duality of structure - the specific demands of his work - and his agency - his interaction with the alerts in the EMOS - both governed his act of utilising the system and the extent and character of that utilisation.

> *"[...] it can depend on the nature of the alerts, how urgent it seems, and the special circumstances of my workplace [...] some things might actually get side-lined for a few weeks if they're not clinically urgent, but [...] the next time I catch up with my paperwork then I'll dig up that alert[...]and review the situation" (GP4)*

For the CCG pharmacist, undertaking medication reviews in care homes the system changed the way they worked because *"if necessary if there's something that comes up on Eclipse whilst we're there we can, rather than having to go back to the surgery first, check it and then make a decision"* (CCGP1). In this way the technology shaped their actions. Furthermore the technological structures in the EMOS and the internal structures led to new shared decision making, use and outcome.

> *"We can look on Eclipse and most of the time it's on Eclipse and we can answer the question there and then. For example, we had a patient who was on Memantine, who was a really not very well gentleman, [...] so we phoned the GP straightaway."(CCGP1)*

Perceptions of the EMOS as requiring technical competence

The EMOS was conceptualised as a "clever" system that could conduct complex searches, but would require technical knowledge on the part of users in order to do so. This allowed for this GP's limited use of the system when combined with an understanding of his own abilities to use the system;

> *"That's how I become accustomed to doing things, which is perhaps why I then don't use Eclipse, because I do think I might not have the ability and the power of making the use of a more powerful tool. But, perhaps I have also then learned useful habits with the old fashioned way." (GP4)*

For peer review only - http://bmjopen.bmj.com/site/about/guidelines.xhtml

Non-use of the system was associated with the cognitive and physical demands associated with using the EMOS and finding time to learn how to get the best out of it. This further conceptualised the system as complex requiring time, training and *"proper teaching"* (GPM2) to gain the expertise required to utilise it.

> *"And if you had the time to log into it and go oh, what does this do? What does that do? […] You train your audit clerk who runs all sorts of searches and does all sorts of audit work, you could have the time to show her and teach her,[…] I'd love to have the time to tinker with,(the system).[…] You'd need time to play with it and time to…proper teaching, proper (training) showing us what it does" (GPM2)*

The conceptualisation of the EMOS as requiring technical competence was related to structures embedded within the technology that allowed for or constrained its use. This could either empower users and thus facilitate further use or could undermine that agency.

> *"And also I'm computer literate and I can work out, I can problem solve because I'm reasonably well educated, if you were talking about average population here, they would either give up, they would probably have given up when they couldn't log in" (Pt2)*

This service user conceptualises the system as difficult and one that required her abilities as a "*computer literate"* to use it. This required an interaction of her capabilities and the structures within the system to engage with it and difficulties with logging in was perceived to be a potential constraint for other users.

Interactions and relationships: Individual, shared and collective use of the technology

There were variations in the ways the technology was used within collaborative networks of social relations. Different general practice staff took responsibility for using the technology; use depended upon shared or collective roles, or upon a hierarchical allocation of access.

For service users, using the technology was determined by networks of social relations. This was expressed as having support from medical professionals to understand the system.

For peer review only - http://bmjopen.bmj.com/site/about/guidelines.xhtml

"But I think the important thing is before you sort of almost start using it, you do need that kind of intervention from a medical practitioner in some way to actually help you with the things you need to know" (Pt1)

Within general practices, there was variation in who took responsibility for the EMOS. On receiving an alert through the system one practice manager would then *"pass it on to the GP and get them to respond to me" (GPM 2)* and that *"the doctors don't access it at all. […] I'm the only one that, yeah, has anything to do with it."(GPM 2)* Another remarked:

"I get the alert the same way through the email, I identify the patient [...] then mine goes to the GP. But the GP actions it, I don't have any more responsibility for it after that […] They go into Eclipse, they do it, […] my job is just to literally give them the information and they do the rest." (GPM1)

Such variation was driven by the conventions and norms associated with work practices. In different general practices individuals were assigned to different roles and responsibilities often based on what worked best for the practice.

"one of the GPs has been nominated within our practice to take that lead in the same way that we break our workload down in other areas; you be the lead for this and tell us if there's anything we all need to know and share the workload." (GPM4)

The allocation of access to the EMOS limited its use. Community pharmacists did not have access to the system. Perceived social norms were seen as *"historically a barrier"* (CP2) that perpetuated that lack of access. Community pharmacists attributed this barrier to GPs seeing themselves as "*as the custodians of the patient record"* (CP2).

"I think there always has been a conflict because GPs often see themselves as the custodians of the patient record and even though the information in that patient record, even abbreviated information is incredibly useful for community pharmacists, they've never successfully managed

For peer review only - http://bmjopen.bmj.com/site/about/guidelines.xhtml

to allow us access and this is going back to EPS [Electronic Prescription Service], this is what EPS promised and it's never happened." (CP2)

There was a perception that the system was a tool for the CCG. This differential access meant that the system had not been used in some general practices. There were however perceptions that the system had *"evolved"*.

"I think that's what it was [...] originally purchased…or the agreement with Eclipse was originally for the meds management team to use it as a tool for them [...] And I think Eclipse has evolved since that happened [...] And I don't think any of us have kept up with how Eclipse has evolved and what else it can now do."(GPM3)

Such changes were related to social norms around ownership and conventions concerning how the system would be used; centrally by the CCG to look at prescribing patterns across practices, and by individual practices of their own prescribing audits. As the system evolved there were perceptions that it could do more. In this way, perceptions of the technological structures and material properties of the technology drove the ambitions of some users to learn more about the potential uses of the system which opened up access to different users.

DISCUSSION

The adoption and implementation of the EMOS was dependent upon a dynamic mix of external structures, internal structures and the material properties embedded in the technology. External infrastructures, the motivations of users and the material properties of the EMOS facilitated information gathering. Perceiving the system as new could lead to resistance and the maintenance of habitual behaviours. Use was dependent upon interactions and relationships between users. Use could be further constrained by conceptualising the system as requiring technical competence.

SST proposes that in order to act, agents draw upon internal structures. These internal structures include dispositions and knowledge of the "strategic terrain" of external structures [29]. It has been suggested that to understand the implementation and adoption of IT from a SST standpoint it is important to

For peer review only - http://bmjopen.bmj.com/site/about/guidelines.xhtml

understand the context in which the IT is being introduced, the networks of people and technologies, the dispositions of actors in those networks, the material properties of the technology and how those shape human action [29, 32]. In the present study, the contextual background was shaped by policy relating to medication safety and the requirement to benchmark against national prescribing and safety targets. CCG managers' knowledge of the external structures relating to that policy background, and their own skills and ambitions, led to actions around the monitoring of prescribing behaviours across the CCG area. This was facilitated by material properties in the system. The outcomes of the monitoring actionswere not just that prescribing data was gathered and reported to other institutions but that the external structures, the dispositions of the CCG managers and the material properties of the system allowed for governance and monitoring of clinicians behaviours through tracking engagement with the system and processes of persuasion and reward. This could therefore have been said to reinforce hierarchical relationships between the CCG and local GPs. Hence, the use of the system created new internal structures concerning such social rules and conventions. Similarly, in previous literature, information systems have been associated with enabling managers to capture information, place local clinicians under surveillance and make their actions calculable [39]. Furthermore, an effect of such surveillance is for individuals to adapt their own behaviour to ensure they act legitimately [40].

Previous literature has established the role of social, organisational and work practices in the adoption of IT, [16,1] others have focused upon functionality of design and tailoring to users [18, 41] or upon top-down implementation [41, 42]. Other research has indicated that emphasis upon training might also construct end-users as the problem [43]. With notable exceptions [29, 33, 34], much of this earlier literature has highlighted the importance of work practices and how technology needs to be embedded into pre-existing routines, but has not seen these dynamically linked to wider contexts particularly in the context of medication safety in primary care. In this study we found that key agents in the network either resisted or sustained use of the system. GPs saw the system as new and unnecessary and not compatible with existing workplace routines. There were also differences in agents responses to the material properties in the system where these were seen as facilitating use by some agents and by others as a barrier to use because the material properties of the system were perceived as to make it to difficult to

For peer review only - http://bmjopen.bmj.com/site/about/guidelines.xhtml

use. SST enabled us to understand these dispositional behaviours in relation to social structures particularly pre-existing routines, work practices and social norms. In previous research there has been a focus upon interoperability, work practices and system usability suggesting that poor adoption of IT is related to users or the system [19, 41]. This misses how interactions and relationships between contexts, users and the technology might work and how the implementation of IT is a social practice [39]. In this study, these networks of social relations impacted upon the use of the system.

Implications of the findings

SST would argue that individual agency is dependent upon knowledge of rules and conventions. Primary care settings are governed by institutional norms, measures, rules and traditions, habits and behaviours [29, 44]. Some of these are embedded in local rules and conventions associated with the different working dynamics of individual practices, while others are found in regulations and governance associated with wider economic and institutional contexts [29, 45]. Using SST in this way may be particularly valuable in primary care research, as general practices operate with their own organizational culture and dynamic which may well lead to marked differences in working practices and structure [46].

This study highlights how healthcare IT interventions are implemented and adopted in a complex social and organisational context. Interventions that are top down and perceived as tools of managerial control are less likely to be effective than those that take into consideration existing local practices and the ambitions and attitudes of those who will use the technology.

Strengths and limitations

Much of the previous literature on interventions to improve medication safety has focused upon secondary healthcare settings, and electronic audit and feedback systems of the kind examined in this study are under-researched in primary care. A particular strength of this study is the use of strong structuration theory which was found to be a useful theoretical approach to studying the implementation and adoption of the EMOS in a primary care setting. In applying this theoretical approach we were able to see the differences in motivations, ambitions, aims and attitudes of different actors from different

For peer review only - http://bmjopen.bmj.com/site/about/guidelines.xhtml

stakeholder groups towards the IT intervention. Strong structuration theory could also reveal the complex contextual background in which the EMOS was implemented and revealed how the implementation was informed by wider contexts. Hence we were able to understand that the successful adoption of the EMOS was not merely dependent upon agents but upon the complex terrain in which it was implemented. Previous studies using this approach have focused upon large national IT projects where institutional contexts might be considered to have more impact [32, 47]. We found however that in a smaller scale project, wider policy institutional contexts did impact upon the implementation and adoption of the IT for example through the CCG's response to the requirements of national policies. In this way the use of the system depended on other factors alongside the dispositions of the users.

There are several limitations to this work which present further opportunities for future research examining the adoption and implementation of electronic audit and feedback systems to improve medication safety in primary care settings. It has been suggested that studies such as these explore wider social contexts through analysis of background data and through ethnographic observation [35, 48].Though we conducted one observation with a CCG pharmacist this was only as an extension of the interview with that participant to elicit some further understanding of how they used the EMOS. A number of naturalistic observations would have been useful in unpicking contexts and agents' choices and actions in using the system.

CONCLUSION

Our study examines the implementation and adoption of an IT system for medicines optimisation in primary care. It was found that the dynamic combination of external, internal and technological structures impacted upon the adoption and implementation of the system. Information technology interventions for medicines optimisation should consider how utilisation may depend on a combination of the infrastructure within primary care, social structures embedded in the technology and the conventions, norms and dispositions of those utilising it.

Figures

For peer review only - http://bmjopen.bmj.com/site/about/guidelines.xhtml

Figure 1. Strong structuration theory incorporating a technology dimension (adapted from Stones, 2005).

Figure legend: Strong structuration theory incorporating a technology dimension (adapted from Stones, 2005).Trisha Greenhalgh, Rob Stones ***Theorising big IT programmes in healthcare: Strong structuration theory meets actor-network theory*** *Social Science & Medicine, Volume 70, Issue 9, 2010, 1285–1294*

For peer review only

Abbreviations

EMOS: Electronic medicines optimisation system; CCG: Clinical commissioning group. IT: Information technology; SST: Strong Structuration Theory; GP: General practitioner; NHS: National Health Service.

Acknowledgments

We are grateful to all participants who kindly gave their time. We would also like to acknowledge the assistance given by the NIHR Clinical Research Network.

Contributors

All authors were involved in the design of this study. MJ led on recruitment of participants, data collection, analysis of the data and drafting of the article. RLH helped co-facilitated focus groups. DLP, RLH, SR, AJA and DMA also made contributions to analysis and interpretation. All authors revised the article critically and approved the final version to be published.

Funding

This research was funded by the National Institute for Health Research (http://www.nihr.ac.uk) through the Greater Manchester Primary Care Patient Safety Translational Research Centre (NIHR GM PSTRC), grant number gmpstrc-2012-1.

For peer review only - http://bmjopen.bmj.com/site/about/guidelines.xhtml

Disclaimer

The views expressed are those of the author(s) and not necessarily those of the NHS, the NIHR or the Department of Health**.**

Competing interests

The authors declare they have no competing interests.

Ethical approval and consent to participate

All participants gave informed written consent to take part in the study, and for the interviews and focus groups to be audio recorded and transcribed verbatim . Ethical approval for the study was granted by the NHS National Research Ethics Service (reference 14/NW/0113)

Availability of data

Data cannot be shared because participants did not consent to this. In addition since this is a small case study, involving small numbers of participants, there is a possibility that material in the transcripts could identify participants.

References

1.HSCIC. Prescriptions Dispensed in the Community: England 2004-2014. London: Health and Social Care Information Centre; 2015.

2. Naylor C, Imison C, Addicott C, et al. Transforming our health care system: Ten priorities for commissioners. London: The King's Fund; 2015

3. Guthrie B, Makubate B, Hernadez-Santiago V, et al. The rising tide of polypharmacy and drug-drug interactions: population database analysis. 1995-2010 *BMC Med.* 2015; 13:74.

For peer review only - http://bmjopen.bmj.com/site/about/guidelines.xhtml

4. Avery AJ, Ghaleb, M, Barber N et al. The prevalence and nature of prescribing and monitoring errors in English general practice: a retrospective case note review. *Br J Gen Pract.* 2013; 63(613): e543-e553

5. Avery AJ, Sheikh A, Hurwitz B et al. Safer medicines management in primary care. *Br J Gen Pract.* 2002; 52 Suppl: S17-S22.

6. Akbarov A, Kontopantelis E, Sperrin M, et al. Primary care medication safety surveillance with integrated primary and secondary care electronic health records: a cross-sectional study. *Drug Saf.* 2015; 38:7: 671-682

7. Stocks SJ, Kontopantelis E, Akbarov A, et al. Examining variations in prescribing safety in UK general practice: cross sectional study using the Clinical Practice Research Datalink. *BMJ* 2015;351:h5501

8. Taché VS, Sönnichsen A, Ashcroft DM. Prevalence of adverse drug events in ambulatory care: a systematic review. *Ann Pharmacother*. 2011; 45; 977-989.

9. Kongkaew C, Hann M, Mandal J, et al. Risk factors for hospital admissions associated with adverse drug events. *Pharmacotherapy*. 2013; 33(8); 827-37.

10. Department of Health, *Building a Safer NHS: Improving Medication Safety* London: The Stationery office 2004.

11. NPSA, *Seven steps to patient safety for primary care: The full reference guide.* London: The National Patient Safety Agency 2006

12. Duerden, M, Avery, AJ, and Payne R. *Polypharmacy and medicines optimisation: Making it safe and sound.* London: The Kings Fund 2013

13. Department of Health, *An Organisation With a Memory: Report of an expert group on learning from adverse events in the NHS*. London; The Stationery Office 2000.

For peer review only - http://bmjopen.bmj.com/site/about/guidelines.xhtml

14. Isle of Wight NHS Trust – Health Services – Medicines Management team http://connect.iow.nhs.uk/index.asp?record=1492 accessed January 2017.

15. Zahabi M, Kaber, DB, Swangnetr M. Usability and Safety in Electronic Medical Records Interface Design: A Review of Recent Literature and Guideline Formulation. *Hum Factors.* 2015; 57(5); 805-34.

16. Hayward J, Thomson F, Milne H, et al. "Too much, too late": mixed methods multi-channel video recording study of computerized decision support systems and GP prescribing. *J Am Med Inform Assoc*. 2013; e76-e84

17. Burgin A, O'Rourke R, Tully MP. Learning to work with electronic patient records and prescription charts: experiences and perceptions of hospital pharmacists. *Res Soc Adm Pharm.* 2014; 10(5); 741-55.

18. Harvey J, Avery AJ, Hibberd R, et al. Meeting user needs in national healthcare systems: lessons from early adopter community pharmacists using the electronic prescriptions service. *BMC Med Inform Decis Mak.* 2014; 14,16

19. Garfield S, Hibberd R, Barber N. English community pharmacists' experiences of using electronic transmission of prescriptions: a qualitative study. *BMC Health Serv Res.* 2013; 13; 343

20. Swinglehurst D, Greenhalgh T, Russell J,et al. Receptionist input to quality and safety in repeat prescribing in UK general practice: ethnographic case study. *BMJ* 2011;343:d6788.

21. Peiris D, Usherwood T, Weeramanthri T, et al. New tools for an old trade: a socio-technical appraisal of how electronic decision support is used by primary care practitioners. *Socio Health Illn.* 2011; 33(7): 1002-1018

22. Timmermans S, Berg M. The practice of medical technology. *Socio Health Illn.* 2003; 25:97-114

23. Clegg C. Sociotechnical principles for system design. *Appl Ergon*. 2000; 31: 463-477

For peer review only - http://bmjopen.bmj.com/site/about/guidelines.xhtml

24. Berg M. Problems and promises of the protocol. *Soc Sci Med.*1997; 44(8):1081-8

25. Clegg C. Shepherd C. "The biggest computer programme in the world... ever!": time for a change of mindset. *J Inform Technol*. 2007; 22: 212-221

26. Aarts A. Sociotechnical perspective of electronic prescribing in Tully MP, Dean Franklin B (Eds) Safety in Medication Use. Boca Raton FL: CRC Press 2015

27. Berg M. Implementing information systems in health care organizations: myths and challenges. *Int J med inform*. 2001; 64: 143-156

28. Oroviogoicoechea C, Watson R. A quantitative analysis of the impact of a computerised information system on nurses clinical practice using a realistic evaluation framework. *Int J med inform*. 2009; 78:839-849

29. Greenhalgh T, Swinglehurst D, Stones R. Rethinking resistance to big IT: a sociological study of why and when healthcare staff do not use nationally mandated information and communication technologies. *Health Serv Deliv Res*. 2014; 2(39)

30. Stones R. Structuration Theory. Basingstoke: Palgrave-Macmillan; 2005.

31. Giddens A: The constitution of society: outline of the theory of structureBerkeley, CA: University of California Press; 1984

32. Greenhalgh T, Stones R. Theorising big IT programmes in healthcare: strong structuration theory meets actor–network theory. *Soc Sci Med* 2010;70:1285–94.

33. Hinder S, Greenhalgh T. "This does my head in" Ethnographic study of self-management by people with diabetes *BMC Health Serv Res* 2012; 1:83

For peer review only - http://bmjopen.bmj.com/site/about/guidelines.xhtml

34 . Greenhalgh T, Stones R, Swinglehurst D. Choose and Book: a sociological analysis of ‘resistance’ to an expert system, *Soc Sci Med* 2014; 104:210-19

35 .Greenhalgh T, Swinglehurst D. Studying technology uses as a social practice: the untapped potential of ethnography. *BMC Med*. 2011; 9:45

36 Eclipse Solutions. Eclipse Live. https://www.eclipsesolutions.org/EclipseInfo/AboutEclipse/ Accessed August 2014

37. King, N. ‘Doing template analysis’, in G.Symon and C.Cassell (eds.) Qualitative Organizational Research: Core Methods and Current Challenges*.* London: Sage 2012.38.

38. Hardcastle MR, Usher KJ, Holmes CA, An overview of structuration theory and its usefulness in nursing research. *Nurs Philos* 2005; 6: 223-234

39. Greenhalgh T, Russell J. Why do evaluations of eHealth programs fail? An alternative set of guiding principles. *PLOS Med*. 2010: 7:e1000360

40. Doolin B. Power and resistance in the implementation of a medical management information system. *Inform Syst J* 2004;14:343–62.

41. Cresswell KM, Worth A, Sheikh A. Integration of a nationally procured electronic health record system into user work practices *BMC Med Inform Decis Mak*. 2012;12:15

42. Sheikh A, Cornford T, Barber N, et al. Implementation and adoption of nationwide electronic health records in secondary care in England: final qualitative results from prospective national evaluation in "early adopter" hospitals. *BMJ*. 2011; 343:d6054

43. Waterson P. Health information technology and sociotechnical systems: A progress report on recent developments within the UK National Health Service (NHS). *Appl Ergon*. 2014; 45: 150-161

For peer review only

44. Daker-White G, Hays R, McSharry J, et al. Blame the patient, blame the doctor? A meta-synthesis in qualitative studies of patient safety in primary care. *PLOS One*. 2015; 10(8), e0128329

45. Winthereik BR, van der Ploeg I, Berg M. The electronic patient record as a meaningful audit tool: accountability and autonomy in general practitioner work. *Sci Technol Hum Values* 2007;32:6–25.

46. Esmail, A. *Measuring and monitoring safety: a primary care perspective.* London: The Health Foundation 2013.

47. Cresswell KM, Bates DW, Sheikh A. Ten key considerations for the successful implementation and adoption of large-scale health information technology. *J Am Med Inform Assoc.* 2013; 0, 1-5

48. Swinglehurst D, Greenhalgh T, Myall M, et al. Ethnographic study of ICT-supported collaborative work routines in general practice. *BMC Health Serv Res*. 2010;10:348

For peer review only

For peer review only - http://bmjopen.bmj.com/site/about/guidelines.xhtml

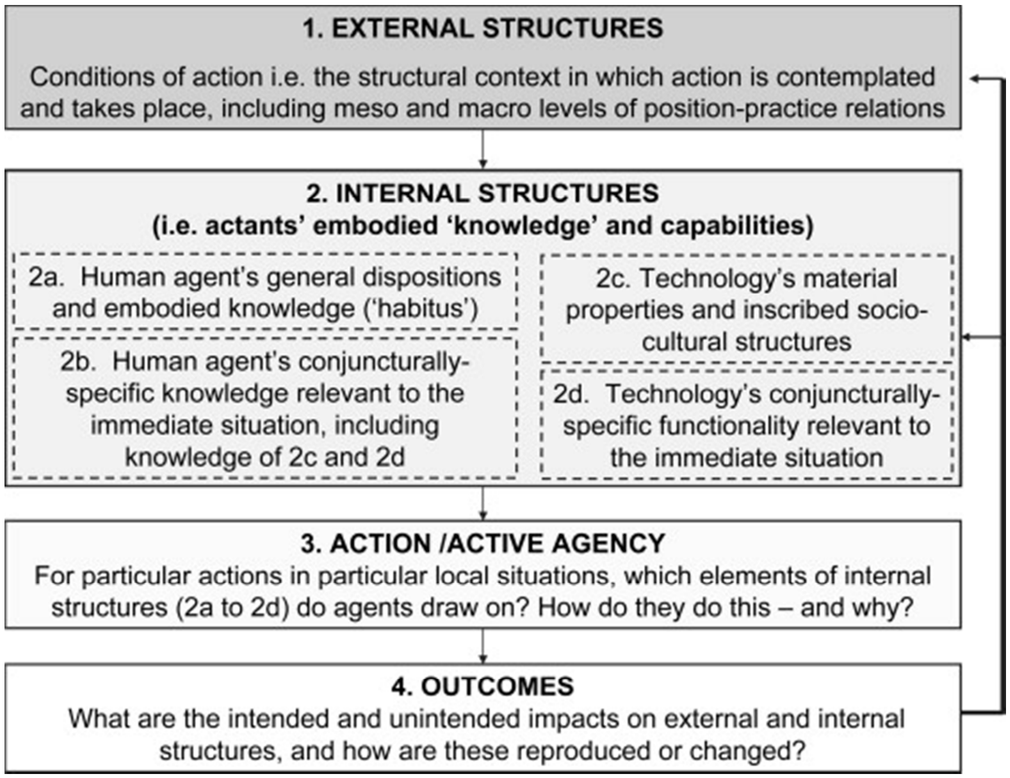

120x92mm (113 x 113 DPI)

For peer review only

Understanding the implementation and adoption of an information technology intervention to support medicines optimisation in primary care: qualitative study using strong structuration theory

COREQ checklist

Note: in order to minimize the length of the manuscript, some of the details on the checklist (marked '*') are not included in the manuscript.

Guide question		*Response*	*Page number in manuscript*
1	Interviewer/facilitator	MJ conducted the interviews. MJ and RLH facilitated the focus groups	9
2	Credentials	MJ holds an MSc in Health Psychology<br>RLH holds a PhD in Medic8ines safety in Primary care	9
3	Occupation	MJ : Research Associate in medication safety;<br>RLH: freelance research pharmacist	9
4	Gender	MJ male; RLH female	9
5	Experience and training	Both researchers have previous experience of undertaking qualitative research in healthcare at PhD and postdoctoral level	9
6	Relationship established	The researchers were not known to the participants prior to the study	9
7	Participant knowledge of the researcher	Participants were made aware of the reasons for doing the research via the information which was sent to the participant prior to the interview	*
8	Researcher characteristics	The researchers had identified the study topic as part of larger programmes of work in their research groups, medication safety in primary care.	9
9	Methodological orientation and theory	Strong structuration theory. The analysis was thematic using template analysis.	5-6, 10
10	Sampling	Individual participants were recruited on a purposive basis via the study CCG or through community pharmacy networks. All participants were chosen to fit the sampling frame (people within the CCG's geographical area who represented the stakeholder groups: pharmacists, doctors, general practice managers and patients)	8-9
11	Method of approach	Participants were approached by telephone or email	9
12	Sample size	19 participants	8-9
13	Non-participation	A number of possible participants were approached but declined to participate. Predominantly this was for reasons of time, workload or lack of use of the system. These included 2 pharmacist technicians, 2 GPs, 2 community pharmacists and 8 general practice managers.	*
14	Setting of data collection	Four interviews were conducted by telephone and one at the CCG offices, the focus groups were conducted at the CCG offices or at a local hotel.	9
15	Presence of non-participants	No non-participants were present	9

For peer review only

16	Description of sample	See Table 1 of the main manuscript	8
17	Interview guide	In the interviews and focus groups we explored experiences of working with the EMOS, perceptions of the system, benefits and drawbacks, the organisational structures and roles required for its use and the circumstances under which it was considered most effective. No pilot testing was undertaken due to the small scale nature of the study, the timescale of the study and the difficulties of recruitment.	9
18	Repeat interviews	None -One GP was interviewed and participated in a focus group	9
19	Audio/visual recording	Audio recording only, with consent from the participant	9
20	Field notes	None	*
21	Duration	The interviews lasted between 20 and 50 mins. The focus groups lasted between 57-112 mins.	9
22	Data saturation	Data collection continued until saturation was reached and no new themes emerged from the interviews and focus groups.	9
23	Transcripts returned	No transcripts were returned to participants	*
24	Number of data coders	MJ coded the data but regular discussions codes were held with all authors. Coding template reviewed by MJ and DLP	10
25	Description of the coding tree	A coding tree description is not given but details on a priori codes is included	10
26	Derivation of themes	A priori thematic codes were applied to the data and new themes emerged from the data. This is described in the analysis section	10
27	Software	QSRNvivo 10 software was utilised to manage the data	10
28	Participant checking	No	*
29	Quotations presented	Please see the results section of the manuscript	10-17
30	Data and findings consistent		10-17
31	Clarity of major themes		10-17
32	Clarity of minor themes		10-17

For peer review only

BMJ Open

Understanding the implementation and adoption of an information technology intervention to support medicines optimisation in primary care: qualitative study using strong structuration theory

Journal:	*BMJ Open*
Manuscript ID	bmjopen-2016-014810.R2
Article Type:	Research
Date Submitted by the Author:	24-Mar-2017
Complete List of Authors:	Jeffries, Mark; University of Manchester, Centre for Pharmacoepidemiology and Drug Safety,School of Health Sciences; University of Manchester, NIHR Greater Manchester Primary Care Patient Safety Translational Research Centre<br>Phipps, Denham; University of Manchester, Centre for Pharmacoepidemiology and Drug Safety, School of Health Sciences; University of Manchester, NIHR Greater Manchester Primary Care Patient Safety Translational Research Centre<br>Howard, Rachel; Esplanade Surgery<br>Avery, Anthony; University of Nottingham, School of Medicine<br>Rodgers, Sarah; University of Nottingham, Division of Primary Care<br>Ashcroft, Darren; University of Manchester, Centre for Pharmacoepidemiology and Drug Safety, School of Health Sciences; University of Manchester, NIHR Greater Manchester Primary Care Patient Safety Translational Research Centre
**Primary Subject Heading**:	General practice / Family practice
Secondary Subject Heading:	Qualitative research, Sociology, Health services research
Keywords:	Prescibing, Information technology < BIOTECHNOLOGY & BIOINFORMATICS, PRIMARY CARE, Strong Structuration Theory, Medicines optimisation

SCHOLARONE™
Manuscripts

Understanding the implementation and adoption of an information technology intervention to support medicines optimisation in primary care: qualitative study using strong structuration theory

Corresponding author:-

Mark Jeffries, Centre for Pharmacoepidemiology and Drug Safety, School of Health Sciences, Faculty of Biology, Medicine and Health, University of Manchester, Manchester, United Kingdom;

mark.jefferies@manchester.ac.uk

0161 275 3680

Denham L Phipps [1,2]

Rachel L Howard [3]

Anthony J Avery [4]

Sarah Rodgers [4]

Darren M Ashcroft [1,2]

[1]Centre for Pharmacoepidemiology and Drug Safety, School of Health Sciences, Faculty of Biology, Medicine and Health ,University of Manchester, Manchester, United Kingdom

[2]NIHR Greater Manchester Primary Care Patient Safety Translational Research Centre, University of Manchester, Manchester Academic Health Sciences Centre (MAHSC), United Kingdom

[3] Research Pharmacist, Isle of Wight, United Kingdom

[4] Division of Primary Care, University of Nottingham, Nottingham, United Kingdom

Word count: 5684

For peer review only

For peer review only - http://bmjopen.bmj.com/site/about/guidelines.xhtml

ABSTRACT

Objectives

Using strong structuration theory, we aimed to understand the adoption and implementation of an electronic clinical audit and feedback tool to support medicines optimisation for patients in primary care.

Design

A qualitative study informed by strong structuration theory. The analysis was thematic, using a template approach. An *a priori* set of thematic codes, based upon strong structuration theory, was developed from the literature and applied to the transcripts. The coding template was then modified through successive readings of the data.

Setting

Clinical commissioning group in the South of England.

Participants

Four focus groups and five semi-structured interviews were conducted with eighteen participants purposively sampled from a range of stakeholder groups (general practitioners, pharmacists, patients and commissioners).

Results

Using the system could lead to improved medication safety but use was determined by broad institutional contexts, by the perceptions, dispositions and skills of users and by the structures embedded within the technology. These included: perceptions of the system as new and requiring technical competence and skill; the adoption of the system for information gathering; and interactions and relationships that involved individual, shared or collective use. The dynamics between these external, internal and technological structures affected the adoption and implementation of the system.

For peer review only - http://bmjopen.bmj.com/site/about/guidelines.xhtml

Conclusions

Successful implementation of information technology interventions for medicines optimisation will depend on a combination of the infrastructure within primary care, social structures embedded in the technology and the conventions, norms and dispositions of those utilising it. Future interventions, using electronic audit and feedback tools to improve medication safety, should consider the complexity of the social and organisational contexts and how internal and external structures can impact upon the use of the technology in order to support effective implementation.

Strengths and limitations of this study

- This is the first study to explore the implementation of electronic audit and feedback systems to improve medication safety in primary care using strong structuration theory.
- Strong structuration theory was found to be particularly valuable for unpicking why the system was used and the different motivations, ambitions, aims and attitudes of a range of stakeholders.
- This was an exploratory study that relied mainly upon interview and focus group data from a number of key stakeholders located in one clinical commissioning group in England.
- Additional insights may have been gained by undertaking ethnographic observation to discover exactly the ways people utilised the Electronic Medicines Optimisation System (EMOS)

INTRODUCTION

Prescribing of medicines to patients is the most common clinical intervention in primary care. However, with high volumes of medicines prescribed in primary care [1], the prevalence of repeat prescribing and the increased burden and complexity of multimorbidity and related polypharmacy [2,3], there is an increased likelihood that prescribing or monitoring errors can occur [4,5]. Recent studies using prescribing safety indicators to investigate the prevalence of hazardous prescribing in

For peer review only - http://bmjopen.bmj.com/site/about/guidelines.xhtml

primary care found 5.2 to 5.5% of patients to be at risk of potentially hazardous prescribing and 7.6 to 11.8% of patients not receiving recommended monitoring tests [6,7]. Some medication errors may not lead to harm; however, approximately 13% of patients have experienced an adverse drug event after receiving prescription medication in primary care, and many of those have been serious enough for patients to seek medical assistance at hospital [8, 9]. The monitoring of patients in receipt of prescription medication is therefore considered important in order to avoid potentially serious adverse drug events.

In the UK, national and local policies have set out recommendations for medication safety improvement [10-12]. "An Organisation with a Memory" [13] set out the necessity for the establishment of a patient safety culture within healthcare organisations. This emphasised the importance of organisational practices. Policy has also set out how the utilisation of information technology presents opportunities to fulfil medication safety requirements and that the contribution of information systems should be maximised [11]. This was further enhanced by Department of Health recommendations in the report “Building a Safer NHS for patients: Improving Medication Safety” [10],which recommended that steps to safer prescribing may include the implementation of effective IT systems particularly those systems that might highlight and give warnings to medical staff of prescription errors. Similar recommendations have suggested there is a need to develop systems that optimise the use of medicines and that this might include improved electronic decision support for clinicians [12]. Locally, the Clinical Commissioning Group (CCG) that formed the setting for this study operated a prescribing incentive scheme designed to improve the quality of prescribing, respond to the requirements of national guidelines, and reduce excessive prescribing and costs, which was incentivised by small financial rewards for general practices [14].

Healthcare information technology (IT) systems may be useful for monitoring medication usage. However, the implementation of such IT has not always been successful, with technology being resisted, not used effectively or used differently than was planned. Previous research has suggested that reasons for this might reside in the design and functionality of the technology. Poorly designed or implemented IT systems have been seen to create cognitive overload [15], and disrupt workflow [16].

For peer review only - http://bmjopen.bmj.com/site/about/guidelines.xhtml

For peer review only

Furthermore, IT systems may be utilised in ways unintended by developers, either to overcome problems with design or as new uses for the technology become apparent [17-19]. However, such tailoring of systems suggests a dynamic where implementation actually involves interpretation and adaptation of systems to fit existing work practices, or changes to work practices in order to adapt to the new system [20-25]. In other words, the success or failure of an IT implementation could be seen as being shaped by interactions between the technology, the users and social and organisational processes [26-28].This sociotechnical view, rather than focusing only upon the functionality of systems, takes into account the complex nature of healthcare and the cultural, social and organisational aspects of the workplaces [22-29].

Strong structuration theory (SST) has been proposed as a way of examining these sociotechnical aspects of healthcare IT implementation [30]. It is based on Giddens' structuration theory, which proposed a relationship between structures (such as social norms, political and economic institutions) and agency (people's actions and choices) [31]. According to Stones [30], SST extends this structure - agency relationship to include the following elements (see figure 1):

- *External structures*, which are the physical social or economic context in which action is contemplated. External structures are built through social positions, practices and networks of social relationships [29, 32].These could include hierarchical relationships between employers and employees, professional roles, local and national guidelines, governance measures, regulations, professional codes of practice, as well as local work practices and interactions among groups of stakeholders [30, 32].
- *Internal structures*, which are manifest in two ways. Firstly, as the skills, dispositions, ambitions, attitudes, values, past experiences of actors and ways of viewing the world; Secondly as the actors' knowledge of rules, conventions, obligations and social norms, which may involve partial understandings and past experiences. These inform how one is supposed to act in specific situations in the here and now, based upon the agents understanding of external structures [30, 32-33].

For peer review only - http://bmjopen.bmj.com/site/about/guidelines.xhtml

- *Agency,* which is how and why agents draw upon internal structures to act in particular ways in specific situations [32]
- *Outcome,* which is the way agency impacts on external or internal structures and how they are maintained or changed [32].

Stones and Greenhalgh [32] further explained the role of technology in SST: rather than there being symmetry between technology and human actors, they are instead separate and may act in different ways [32]. Technology incorporates procedures, codes, material properties and standards that can enable or constrain use [19, 32-34]; it is therefore seen as shaping human actions by making certain actions possible [35]. Previous studies [29, 34] suggest that SST can illuminate the implementation and adoption of information technology by understanding how people *"take action with respect to technologies"*; in other words, what people actually do with the systems and to what effect [32]. Strong structuration theory has been previously used to understand the ways a large scale healthcare IT intervention, designed to assist patients and General Practitioners (GPs) to book hospital outpatient appointments, was resisted or adopted [34].

INSERT FIGURE 1

This study uses SST to examine a new electronic medicines optimisation system (EMOS) [36] that was implemented in a primary care locality The EMOS allows different stakeholders - general practitioners, Clinical Commissioning Group (CCG) managers, pharmacists, general practice managers, and patients - access to real time anonymized patient data including medical diagnoses, prescribed medications and laboratory test results. It comprises a secure patient database and a web-based user interface that extracts patient specific data from the general practice clinical record system. The interface provides a number of user functions; these include reviewing a specific patient health record, identifying patients who are at risk of a medication-related adverse event, such as those who are on inappropriate combinations of drugs or who have not received appropriate monitoring and carrying out clinical audits on a subset of patients [36]. The EMOS also allowed clinicians and managers in the health locality to audit prescribing practices across general practices and make

For peer review only - http://bmjopen.bmj.com/site/about/guidelines.xhtml

comparisons against national guidelines. Patients have access to the system through a patient passport which allows them to view their medications and test results. In this context it was felt that SST would unpick the ways in which users of the system drew upon their dispositions, attitudes skills and ambitions and upon their knowledge of and understanding of external structures to engage with the technology. Therefore, we aimed to examine the specific question: “in what ways did external, internal and technological structures impact upon the implementation and adoption of the EMOS?"

METHODS

Study design and setting

Our study used a qualitative design. The study setting was a CCG in the South of England, which was chosen because it was an early adopter of the EMOS and had all general practices signed up to the system. The CCG was relatively small in size (17 separate general practices, and approximately 140,000 patients). Medicines management activities at the CCG were undertaken by three clinical pharmacists (including participants CCGP1 and CCGP2) and two pharmacy technicians. Additionally one GP (participant GP1) operated as prescribing lead for the CCG. In the English National Health Service (NHS), a CCG is a clinically-led statutory NHS body responsible for the planning and commissioning of health care services for their local area, and cover groups of general practices within a local area. The sampling frame was people within the CCG’s geographical area who represented the stakeholder groups. This included doctors, pharmacists, general practice managers and patients.

Understanding the background

Prior to data collection we undertook actions to build a picture of the system and the context in which it was to be used. Authors MJ and RLH were given an overview of the system in a preliminary meeting with the study CCG prior to data collection. In addition MJ visited a separate CCG in the North of England that was utilising the EMOS. Web-based materials relating to the system were read prior to data collection [36].

For peer review only - http://bmjopen.bmj.com/site/about/guidelines.xhtml

1 **Recruitment and data collection**

2 Individual participants were recruited on a purposive basis via the CCG or through community
3 pharmacy networks, to represent the different stakeholder groups (see Table 1).

4 Table 1 – Case study participants

Participants	Role	How they used the EMOS
**Interviews**		
GP1-INT	General Practitioner	In general practice and prescribing lead for the Clinical Commissioning Group (CCG) .Worked with the medicines management team in supporting the adoption of the EMOS by the CCG. Used the EMOS to send alerts to GPs.
GP2	General Practitioner	In general practice and respiratory lead for the CCG. Utilised the EMOS to undertake audits of prescribing relating to respiratory conditions.
GP3	General Practitioner	In general practice
CCGP1 (additional observation as part of interview)	CCG Pharmacist	Utilised the EMOs to undertake medication reviews with care home patients
CCGP2	CCG Pharmacist	CCG medicines management team. Used the EMOS to run audits centrally at the CCG and then alert clinicians locally
**Focus group A - General Practitioners**		
GP4	General Practitioner	In general practice
GP1-FG	General Practitioner	In practice and as prescribing lead for the CCG
**Focus group B – Community Pharmacists**		
CP1	Community Pharmacist	Aware of, but no access
CP2	Community Pharmacist	Aware of, but no access
CP3	Community Pharmacist	Aware of, but no access
CP4	Community Pharmacist	Aware of, but no access
**Focus Group C – Patients**		
Pt1	Patient	Access through patient passport
Pt2	Patient	Access through patient passport
Pt3	Patient	Access through patient passport
Pt4	Patient	Access through patient passport
**Focus Group D - General practice managers**		
GPM1	General Practice Manager	In general practice
GPM2	General Practice Manager	In general practice
GPM3	General Practice Manager	In general practice
GPM4	General Practice Manager	In general practice

For peer review only - http://bmjopen.bmj.com/site/about/guidelines.xhtml

For peer review only

Potential participants were contacted by telephone or email. Five semi-structured interviews (lasting between 20-50 minutes) were conducted with three GPs and two CCG pharmacists, who were known to be using the system, and had specific roles that required the use of the EMOS between August and December 2014. Four homogeneous focus groups (lasting between 57-112 minutes) were also conducted between September and December 2014, each with a specific group of stakeholders: GPs (2); community pharmacists (4); patients (4); and general practice managers (4). No repeat interviews were conducted, although one GP was interviewed and also participated in a focus group. Each focus group was conducted with different a specific type of stakeholder, as this was felt to facilitate free and open discussion.

Topic guides for the interviews and focus groups were developed by reading relevant literature examining the implementation of information technology in healthcare settings. [16, 17, 20, 23, 24]. Both interviews and focus groups were conducted to illicit individual thoughts and opinions and to promote discussion amongst specific homogenous groups of stakeholders. In the interviews and focus groups, we explored experiences of working with the EMOS, perceptions of the system, benefits and drawbacks, the organisational structures and roles required for its use and the circumstances under which it was considered most effective. Data collection continued until saturation was reached and no new themes emerged from the interviews and focus groups. The interviews and focus groups were carried out by a male research associate in medication safety trained and experienced in qualitative health research who holds an MSc in Health Psychology (MJ). The focus groups were co-facilitated by a female freelance research pharmacist experienced in qualitative methodology and with a PhD in Medicines Safety in Primary Care (RLH). The researchers were not known to the participants prior to the study. Four interviews were conducted by telephone and one at the CCG offices. The focus groups were conducted at the CCG offices or at a local hotel solely with the participants, RLH and MJ present. All participants gave written informed consent to take part in the study, and for the interviews and focus groups to be audio recorded and transcribed verbatim. Ethical approval for the study was granted by the Preston NHS Research Ethics Committee (reference 14/NW/0113).

For peer review only - http://bmjopen.bmj.com/site/about/guidelines.xhtml

Analysis

The analysis was thematic, using a template approach [37]. Template analysis involves the summarising of themes through a coding template. Often, template analysis begins with an *a priori* set of themes . New themes are then added, or existing themes revised, as data is iteratively analysed in a process of developing a template [37]. An *a priori* set of thematic codes based upon strong structuration theory was developed from the literature [30, 32, 34, 38]. These included: external structures such as national or local policies, guidelines and governance; interactions, including relationships, conflicts and communication; the internal structures of agents including dispositions, skills, attitudes and cognitive demands; rules and contextuality including routines, social norms and regulations and technological structures including the social structures built into the technology. This set of codes was applied to the transcripts by MJ and documented using the QSR NVivo 10 application. The coding template was then modified through successive readings of the data and discussions with other authors. The template was then internally reviewed for completeness by MJ and DLP (who had independently reviewed all transcripts).

RESULTS

The ways in which the EMOS was implemented and adopted were conceptualised in four broad thematic categories: adoption of the system for information gathering; perceptions of the system as new; perceptions of the EMOS as requiring technical competence; and the interactions and relationships that involved individual or collective use of the technology.

Adoption of the system for information gathering

The EMOS facilitated the efficient acquisition of information relating to the appropriateness of prescribing for individual patients. External structures provided the conditions for the use of the technology; specifically through the requirements of national policies relating to safe medicines use as set down by national governance and guidelines and the CCG's responses to those requirements. The

For peer review only - http://bmjopen.bmj.com/site/about/guidelines.xhtml

CCG was motivated to carry out audits of prescribing, and much of the data extracted through such audits were used to benchmark the CCG against these national policies and targets. This auditing was in turn determined by the policy and institutional climate that required the reporting of such auditing, the setting of certain guidelines and targets, and the adherence to those. This further led to the CCG utilising the technology in a local context to monitor prescribing behaviour in practices in response to local initiatives. External structures such as national or local "initiatives" worked with the internal structures (in this specific instance the motivations of the CCG to report in response to these "initiatives") and the material properties of the technology, to more swiftly identify patients registered with general practices that met the relevant prescribing safety audit. The material properties of the system shaped the ability to conduct extensive searches of electronic health records across multiple general practices in a relatively short space of time. According to the following extract from an interview with a CCG pharmacist the technological structures enabled the collection of data in a more efficient and timely fashion.

> *"[...]it's a way of being able to gather pseudo-anonymised individual patient data and relate it to ideas and thoughts around initiatives that CCG or the medicines management team are looking at that perhaps has been identified or highlighted nationally, or locally and it can all be done relatively quickly within a few seconds if necessary. So you don't have to trawl round 17 different practices"(CCGP2)*

Centrally, in a form of pay for performance initiative, the CCG made the EMOS part of a *"GP incentive scheme to engage with alerts in a meaningful way"* (GP1-INT) and this was conceptualised as *"trying to sort of get some more traction"* (GP1-INT). Guidelines and documents concerning strategies for prescribing framed the possibilities for use "*to actually monitor the progress against a sort of target outcome"* (GP1-INT). The functionality within the EMOS allowed for benchmarking across the CCG. This in turn provided for structures that could be utilised by the CCG to encourage practices to use the system, and an infrastructure that supported their own activities in monitoring prescribing behaviour and to *"reward good prescribing"* (GP1-INT).

For peer review only - http://bmjopen.bmj.com/site/about/guidelines.xhtml

> *"if there are some practices that are demonstrating very good prescribing, then we've picked those out as well and highlighted those to act as a kind of beacon of hope for everybody else". (CCGP2)*

The system also allowed for communication channels and feedback, where contact with practices was through the system or as a result of alerts being sent out by email. Such communication, between the clinicians placed centrally at the CCG and the individual GP practices, enabled the CCG to monitor prescribing as *"a way of looking at the map"* (GP2) as well as the use of the system by *"tracking our advice in those practices"* (GP1-INT). The codes and material properties of the system facilitated monitoring in that logging on to the system indicated engagement with it. This in turn allowed the CCG to further monitor and audit prescribing patterns since they could swiftly see which practices had responded to alerts and *"[could] have some kind of objective measure that [gave them] some idea as to who's perhaps even more engaged than others"* (CCGP2). The ambitions and motivations of the CCG to monitor prescribing acted as an internal structure to work *"very hard to get the uptake of that better"* (CCGP2) and in "*trying to persuade our clinicians to use it so that we get a much more real time feedback."* (CCGP2). Furthermore, this combination of technological infrastructure and the ambitions of the CCG created a new internal structure in the form of a convention for using the system.

> *"(When) the GP logs onto the Eclipse system and there's a little tick box to say patient reviewed [...]. Now some practices are doing that as a regular routine exercise, so that means that tracking our advice in those practices is very easy and what it does allow you to do as well is not to send the same alert out to the same practice again" (GP1-INT).*

In this way there were patterns of agent-technology relationships that reinforced a hierarchical agent - agent relationship within the network. The CCG managers were interacting with the technology to monitor prescribing since engagement at local clinician level with the system was encouraged by the CCG, because it provided further feedback to them, agent-technology relationships could build

For peer review only - http://bmjopen.bmj.com/site/about/guidelines.xhtml

through the system use as new agent -agent relationships between managers centrally at the CCG and local GPs.

Perceptions of the system as new

Using the EMOS was characterised as a new practice that would require new approaches. Resistance towards the system was thus justified by characterising existing behaviours as ingrained. Here, habits and ways of doing things that were presented by one GP as "*the old fashioned way*" (CCGP2), provided for a limited use of the system. One such disposition was around their prescribing habits, which they described as "*conservative*" (CCGP2). This allowed for a limited use of the EMOS, in which most alerts would not require action because prescribing behaviour was already "*protective of patients*"(CCGP2). Similarly, as the following extract illustrates, non-use of the system resulted from habitual accustomed practice of using other systems, pre-existing routines and repetitive ways of doing things.

> *"I think the trouble is Eclipse is another thing you have to log into along with the other 20 things you log into every day, and you're so used to using your other clinical system all the time."(GPM3)*

In a further example of agent-agent relationships associated with the use of the system, the CCG pharmacists were concerned that GPs would otherwise avoid using the system. It was assumed that GPs, in addition to training on the system, needed persuasion in order to "*just [get] them to use it as habit"* (CCGP1).

> *"but we have had a situation where the GP said, oh, I'm not sure if I'll have time to look on Eclipse, but you can't spoon feed them everything"(CCGP1)*

Social structures could shape the ways things were done. Workplace routines and practices, such as the prioritisation of work schedules, acted as constraints or enablers to the use of the new system. Here this GP highlighted contingencies within the structures associated with the *"special circumstances of my workplace"* (GP4) which allowed for a range of actions from side-lining the alert

For peer review only - http://bmjopen.bmj.com/site/about/guidelines.xhtml

through to reviewing the patient. In this way the duality of structure - the specific demands of his work - and his agency - his interaction with the alerts in the EMOS - both governed his act of utilising the system and the extent and character of that utilisation.

> *"[...] it can depend on the nature of the alerts, how urgent it seems, and the special circumstances of my workplace [...] some things might actually get side-lined for a few weeks if they're not clinically urgent, but [...]the next time I catch up with my paperwork then I'll dig up that alert[...]and review the situation" (GP4)*

For the CCG pharmacist, undertaking medication reviews in care homes the system changed the way they worked because *"if necessary if there's something that comes up on Eclipse whilst we're there we can, rather than having to go back to the surgery first, check it and then make a decision"* (CCGP1). In this way the technology shaped their actions. Furthermore the technological structures in the EMOS and the internal structures led to new shared decision making, use and outcome.

> *"We can look on Eclipse and most of the time it's on Eclipse and we can answer the question there and then. For example, we had a patient who was on Memantine, who was a really not very well gentleman, [...] so we phoned the GP straightaway."(CCGP1)*

Perceptions of the EMOS as requiring technical competence

The EMOS was conceptualised as a "clever" system that could conduct complex searches, but would require technical knowledge on the part of users in order to do so. This allowed for this GP's limited use of the system when combined with an understanding of his own abilities to use the system;

> *"That's how I become accustomed to doing things, which is perhaps why I then don't use Eclipse, because I do think I might not have the ability and the power of making the use of a more powerful tool. But, perhaps I have also then learned useful habits with the old fashioned way." (GP4)*

For peer review only - http://bmjopen.bmj.com/site/about/guidelines.xhtml

Non-use of the system was associated with the cognitive and physical demands associated with using the EMOS and finding time to learn how to get the best out of it. This further conceptualised the system as complex requiring time, training and *"proper teaching"* (GPM2) to gain the expertise required to utilise it.

> *"And if you had the time to log into it and go oh, what does this do? What does that do? […] You train your audit clerk who runs all sorts of searches and does all sorts of audit work, you could have the time to show her and teach her,[…] I'd love to have the time to tinker with,(the system).[…] You'd need time to play with it and time to…proper teaching, proper (training) showing us what it does" (GPM2)*

The conceptualisation of the EMOS as requiring technical competence was related to structures embedded within the technology that allowed for or constrained its use. This could either empower users and thus facilitate further use or could undermine that agency.

> *"And also I'm computer literate and I can work out, I can problem solve because I'm reasonably well educated, if you were talking about average population here, they would either give up, they would probably have given up when they couldn't log in" (Pt2)*

This service user conceptualises the system as difficult and one that required her abilities as a "*computer literate"* to use it. This required an interaction of her capabilities and the structures within the system to engage with it and difficulties with logging in was perceived to be a potential constraint for other users.

Interactions and relationships: Individual, shared and collective use of the technology

There were variations in the ways the technology was used within collaborative networks of social relations. Different general practice staff took responsibility for using the technology; use depended upon shared or collective roles, or upon a hierarchical allocation of access.

For service users, using the technology was determined by networks of social relations. This was expressed as having support from medical professionals to understand the system.

For peer review only - http://bmjopen.bmj.com/site/about/guidelines.xhtml

"But I think the important thing is before you sort of almost start using it, you do need that kind of intervention from a medical practitioner in some way to actually help you with the things you need to know" (Pt1)

Within general practices, there was variation in who took responsibility for the EMOS. On receiving an alert through the system one practice manager would then *"pass it on to the GP and get them to respond to me" (GPM 2)* and that *"the doctors don't access it at all. […] I'm the only one that, yeah, has anything to do with it."(GPM 2)* Another remarked:

"I get the alert the same way through the email, I identify the patient [...] then mine goes to the GP. But the GP actions it, I don't have any more responsibility for it after that […] They go into Eclipse, they do it, […] my job is just to literally give them the information and they do the rest." (GPM1)

Such variation was driven by the conventions and norms associated with work practices. In different general practices individuals were assigned to different roles and responsibilities often based on what worked best for the practice.

"one of the GPs has been nominated within our practice to take that lead in the same way that we break our workload down in other areas; you be the lead for this and tell us if there's anything we all need to know and share the workload." (GPM4)

The allocation of access to the EMOS limited its use. Community pharmacists did not have access to the system. Perceived social norms were seen as *"historically a barrier"* (CP2) that perpetuated that lack of access. Community pharmacists attributed this barrier to GPs seeing themselves as "*as the custodians of the patient record"* (CP2).

"I think there always has been a conflict because GPs often see themselves as the custodians of the patient record and even though the information in that patient record, even abbreviated information is incredibly useful for community pharmacists, they've never successfully managed

For peer review only - http://bmjopen.bmj.com/site/about/guidelines.xhtml

to allow us access and this is going back to EPS [Electronic Prescription Service], this is what EPS promised and it's never happened." (CP2)

There was a perception that the system was a tool for the CCG. This differential access meant that the system had not been used in some general practices. There were however perceptions that the system had *"evolved"*.

"I think that's what it was [...] originally purchased…or the agreement with Eclipse was originally for the meds management team to use it as a tool for them [...] And I think Eclipse has evolved since that happened [...] And I don't think any of us have kept up with how Eclipse has evolved and what else it can now do."(GPM3)

Such changes were related to social norms around ownership and conventions concerning how the system would be used; centrally by the CCG to look at prescribing patterns across practices, and by individual practices of their own prescribing audits. As the system evolved there were perceptions that it could do more. In this way, perceptions of the technological structures and material properties of the technology drove the ambitions of some users to learn more about the potential uses of the system which opened up access to different users.

DISCUSSION

The adoption and implementation of the EMOS was dependent upon a dynamic mix of external structures, internal structures and the material properties embedded in the technology. External infrastructures, the motivations of users and the material properties of the EMOS facilitated information gathering. Perceiving the system as new could lead to resistance and the maintenance of habitual behaviours. Use was dependent upon interactions and relationships between users. Use could be further constrained by conceptualising the system as requiring technical competence.

SST proposes that in order to act, agents draw upon internal structures. These internal structures include dispositions and knowledge of the "strategic terrain" of external structures [29]. It has been suggested that to understand the implementation and adoption of IT from a SST standpoint it is important to

For peer review only - http://bmjopen.bmj.com/site/about/guidelines.xhtml

understand the context in which the IT is being introduced, the networks of people and technologies, the dispositions of actors in those networks , the material properties of the technology and how those shape human action [29, 32]. In the present study the contextual background was shaped by policy relating to medication safety and the requirement to benchmark against national prescribing and safety targets. CCG managers' knowledge of the external structures relating to that policy background and their own skills and ambitions led to actions around the monitoring of prescribing behaviours across the CCG area. This was facilitated by material properties in the system. The outcomes from of the monitoring actions were not just that prescribing data was gathered and reported to other institutions but that the external structures, the dispositions of the CCG managers and the material properties of the system allowed for governance and monitoring of clinicians behaviours through tracking engagement with the system and processes of persuasion and reward. This could therefore have been said to reinforce hierarchical relationships between the CCG and local GPs. Hence the use of the system created new internal structures concerning such social rules and conventions. Similarly, in previous literature, information systems have been associated with enabling managers to capture information, place local clinicians under surveillance and make their actions calculable [39]. Furthermore, an effect of such surveillance is for individuals to adapt their own behaviour to ensure they act legitimately [40].

Previous literature has established the role of social, organisational and work practices in the adoption of IT, [16,17] others have focused upon functionality of design and tailoring to users [18, 41] or upon top-down implementation [41, 42]. Other research has indicated that emphasis upon training might also construct end-users as the problem [43]. With notable exceptions [29, 33, 34], much of this earlier literature has highlighted the importance of work practices and how technology needs to be embedded into pre-existing routines, but has not seen these dynamically linked to wider contexts particularly in the context of medication safety in primary care. In this study we found that key agents in the network either resisted or sustained use of the system. GPs saw the system as new and unnecessary and not compatible with existing workplace routines. There were also differences in agents responses to the material properties in the system where these were seen as facilitating use by some agents and by others as a

For peer review only - http://bmjopen.bmj.com/site/about/guidelines.xhtml

barrier to use because the material properties of the system were perceived as to make it to difficult to use. SST enabled us to understand these dispositional behaviours in relation to social structures particularly pre-existing routines, work practices and social norms. In previous research there has been a focus upon interoperability, work practices and system usability suggesting that poor adoption of IT is related to users or the system [19, 41]. This misses how interactions and relationships between contexts, users and the technology might work and how the implementation of IT is a social practice [39]. In this study, these networks of social relations impacted upon the use of the system.

Implications of the findings

SST would argue that individual agency is dependent upon knowledge of rules and conventions. Primary care settings are governed by institutional norms, measures, rules and traditions, habits and behaviours [29, 44]. Some of these are embedded in local rules and conventions associated with the different working dynamics of individual practices while others are found in regulations and governance associated with wider economic and institutional contexts [29, 45]. Using SST in this way may be particularly valuable in primary care research since general practices operate with their own organizational culture and dynamic which may well lead to marked differences in working practices and structure [46].

This study highlights how healthcare IT interventions are implemented and adopted in a complex social and organisational context. Interventions that are top down and perceived as tools of managerial control are less likely to be effective than those that take into consideration existing local practices and the ambitions and attitudes of those who will use the technology.

Strengths and limitations

Much of the previous literature on interventions to improve medication safety has focused upon secondary healthcare settings, and electronic audit and feedback systems of the kind examined in this study are under-researched in primary care. A particular strength of this study is the use of strong structuration theory which was found to be a useful theoretical approach to studying the implementation and adoption of the EMOS in a primary care setting. In applying this theoretical approach we were able

For peer review only - http://bmjopen.bmj.com/site/about/guidelines.xhtml

to see the differences in motivations, ambitions, aims and attitudes of different actors from different stakeholder groups towards the IT intervention. Strong structuration theory could also reveal the complex contextual background in which the EMOS was implemented and revealed how the implementation was informed by wider contexts. Hence we were able to understand that the successful adoption of the EMOS was not merely dependent upon agents but upon the complex terrain in which it was implemented. Previous studies using this approach have focused upon large national IT projects where institutional contexts might be considered to have more impact [32, 47]. We found however that in a smaller scale project, wider policy institutional contexts did impact upon the implementation and adoption of the IT for example through the CCG's response to the requirements of national policies. In this way the use of the system depended on other factors alongside the dispositions of the users.

There are several limitations to this work which present further opportunities for future research examining the adoption and implementation of electronic audit and feedback systems to improve medication safety in primary care settings. It has been suggested that studies such as these explore wider social contexts through analysis of background data and through ethnographic observation [35, 48].Though we conducted one observation with a CCG pharmacist this was only as an extension of the interview with that participant to elicit some further understanding of how they used the EMOS. A number of naturalistic observations would have been useful in unpicking contexts and agents' choices and actions in using the system.

CONCLUSION

Our study examines the implementation and adoption of an IT system for medicines optimisation in primary care. It was found that the dynamic combination of external, internal and technological structures impacted upon the adoption and implementation of the system. Information technology interventions for medicines optimisation should consider how utilisation may depend on a combination of the infrastructure within primary care, social structures embedded in the technology and the conventions, norms and dispositions of those utilising it.

Figures

For peer review only - http://bmjopen.bmj.com/site/about/guidelines.xhtml
For peer review only

Figure 1. Strong structuration theory incorporating a technology dimension (adapted from Stones, 2005).

Figure legend: Strong structuration theory incorporating a technology dimension (adapted from Stones, 2005).Trisha Greenhalgh, Rob Stones **Theorising big IT programmes in healthcare: Strong structuration theory meets actor-network theory** Social Science & Medicine, Volume 70, Issue 9, 2010, 1285–1294

Abbreviations

EMOS: Electronic medicines optimisation system; CCG: Clinical commissioning group. IT: Information technology; SST: Strong Structuration Theory; GP: General practitioner; NHS: National Health Service.

Acknowledgments

We are grateful to all participants who kindly gave their time. We would also like to acknowledge the assistance given by the NIHR Clinical Research Network..

Contributors

All authors were involved in the design of this study. MJ led on recruitment of participants, data collection, analysis of the data and drafting of the article. RLH helped co-facilitated focus groups. DLP, RLH, SR, AJA and DMA also made contributions to analysis and interpretation. All authors revised the article critically and approved the final version to be published.

Funding

This research was funded by the National Institute for Health Research (http://www.nihr.ac.uk) through the Greater Manchester Primary Care Patient Safety Translational Research Centre (NIHR GM PSTRC), grant number gmpstrc-2012-1.

For peer review only - http://bmjopen.bmj.com/site/about/guidelines.xhtml

Disclaimer

The views expressed are those of the author(s) and not necessarily those of the NHS, the NIHR or the Department of Health**.**

Competing interests

The authors declare they have no competing interests.

Ethical approval and consent to participate

All participants gave informed written consent to take part in the study, and for the interviews and focus groups to be audio recorded and transcribed verbatim . Ethical approval for the study was granted by the NHS National Research Ethics Service (reference 14/NW/0113)

Availability of data

Data cannot be shared because participants did not consent to this. In addition since this is a small case study, involving small numbers of participants, there is a possibility that material in the transcripts could identify participants.

References

1.HSCIC. Prescriptions Dispensed in the Community: England 2004-2014. London: Health and Social Care Information Centre; 2015.

2. Naylor C, Imison C, Addicott C, et al. Transforming our health care system: Ten priorities for commissioners. London: The King's Fund; 2015

3. Guthrie B, Makubate B, Hernadez-Santiago V, et al. The rising tide of polypharmacy and drug-drug interactions: population database analysis. 1995-2010 *BMC Med.* 2015; 13:74.

For peer review only - http://bmjopen.bmj.com/site/about/guidelines.xhtml

4. Avery AJ, Ghaleb, M, Barber N et al. The prevalence and nature of prescribing and monitoring errors in English general practice: a retrospective case note review. *Br J Gen Pract.* 2013; 63(613): e543-e553

5. Avery AJ, Sheikh A, Hurwitz B et al. Safer medicines management in primary care. *Br J Gen Pract.* 2002; 52 Suppl: S17-S22.

6. Akbarov A, Kontopantelis E, Sperrin M, et al. Primary care medication safety surveillance with integrated primary and secondary care electronic health records: a cross-sectional study. *Drug Saf.* 2015; 38:7: 671-682

7. Stocks SJ, Kontopantelis E, Akbarov A, et al. Examining variations in prescribing safety in UK general practice: cross sectional study using the Clinical Practice Research Datalink. *BMJ* 2015;351:h5501

8. Taché VS, Sönnichsen A, Ashcroft DM. Prevalence of adverse drug events in ambulatory care: a systematic review. *Ann Pharmacother*. 2011; 45; 977-989.

9. Kongkaew C, Hann M, Mandal J, et al. Risk factors for hospital admissions associated with adverse drug events. *Pharmacotherapy*. 2013; 33(8); 827-37.

10. Department of Health, *Building a Safer NHS: Improving Medication Safety* London: The Stationery office 2004.

11. NPSA, *Seven steps to patient safety for primary care: The full reference guide.* London: The National Patient Safety Agency 2006

12. Duerden, M, Avery AJ, and Payne R.*Polypharmacy and medicines optimisation: Making it safe and sound.* London: The Kings Fund 2013.

13. Department of Health, *An Organisation With a Memory: Report of an expert group on learning from adverse events in the NHS*. London; The Stationery Office 2000.

For peer review only - http://bmjopen.bmj.com/site/about/guidelines.xhtml

14. Isle of Wight NHS Trust – Health Services – Medicines Management team http://connect.iow.nhs.uk/index.asp?record=1492 accessed January 2017.

15 . Zahabi M, Kaber, DB, Swangnetr M. Usability and Safety in Electronic Medical Records Interface Design: A Review of Recent Literature and Guideline Formulation. *Hum Factors.* 2015; 57(5); 805-34.

16. Hayward J, Thomson F, Milne H, et al. "Too much, too late": mixed methods multi-channel video recording study of computerized decision support systems and GP prescribing. *J Am Med Inform Assoc.* 2013; e76-e84

17. Burgin A, O'Rourke R, Tully MP. Learning to work with electronic patient records and prescription charts: experiences and perceptions of hospital pharmacists. *Res Soc Adm Pharm.* 2014; 10(5); 741-55.

18. Harvey J, Avery AJ, Hibberd R, et al. Meeting user needs in national healthcare systems: lessons from early adopter community pharmacists using the electronic prescriptions service. *BMC Med Inform Decis Mak.* 2014; 14,16

19.Garfield S, Hibberd R, Barber N. English community pharmacists' experiences of using electronic transmission of prescriptions: a qualitative study. *BMC Health Serv Res.* 2013; 13; 343

20. Swinglehurst D, Greenhalgh T, Russell J,et al. Receptionist input to quality and safety in repeat prescribing in UK general practice: ethnographic case study. *BMJ* 2011;343:d6788.

21. Peiris D, Usherwood T, Weeramanthri T, et al. New tools for an old trade: a socio-technical appraisal of how electronic decision support is used by primary care practitioners. *Socio Health Illn.* 2011; 33(7): 1002-1018

22. Timmermans S, Berg M. The practice of medical technology. *Socio Health Illn.* 2003; 25:97-114

23. Clegg C. Sociotechnical principles for system design. *Appl Ergon*. 2000; 31: 463-477

24. Berg M. Problems and promises of the protocol. *Soc Sci Med.*1997; 44(8):1081-8

For peer review only - http://bmjopen.bmj.com/site/about/guidelines.xhtml

25 . Clegg C. Shepherd C. "The biggest computer programme in the world... ever!": time for a change of mindset. *J Inform Technol*. 2007; 22: 212-221

26 . Aarts A. Sociotechnical perspective of electronic prescribing in Tully MP, Dean Franklin B (Eds) Safety in Medication Use. Boca Raton FL: CRC Press 2015

27 . Berg M. Implementing information systems in health care organizations: myths and challenges. *Int J med inform*. 2001; 64: 143-156

28 . Oroviogoicoechea C, Watson R. A quantitative analysis of the impact of a computerised information system on nurses clinical practice using a realistic evaluation framework. *Int J med inform*. 2009; 78:839-849

29 . Greenhalgh T, Swinglehurst D, Stones R. Rethinking resistance to big IT: a sociological study of why and when healthcare staff do not use nationally mandated information and communication technologies. *Health Serv Deliv Res*. 2014; 2(39)

30. Stones R. Structuration Theory. Basingstoke: Palgrave-Macmillan; 2005.

31. Giddens A: The constitution of society: outline of the theory of structureBerkeley, CA: University of California Press; 1984

32. Greenhalgh T, Stones R. Theorising big IT programmes in healthcare: strong structuration theory meets actor–network theory. *Soc Sci Med* 2010;70:1285–94.

33. Hinder S, Greenhalgh T. "This does my head in" Ethnographic study of self-management by people with diabetes *BMC Health Serv Res* 2012; 1:83

34. Greenhalgh T, Stones R, Swinglehurst D. Choose and Book: a sociological analysis of 'resistance' to an expert system, *Soc Sci Med* 2014; 104:210-19

For peer review only

For peer review only - http://bmjopen.bmj.com/site/about/guidelines.xhtml

35. Greenhalgh T, Swinglehurst D. Studying technology uses as a social practice: the untapped potential of ethnography. *BMC Med*. 2011; 9:45

36. Eclipse Solutions. Eclipse Live. https://www.eclipsesolutions.org/EclipseInfo/AboutEclipse/ Accessed August 2014

37. King, N. 'Doing template analysis', in G.Symon and C.Cassell (eds.) Qualitative Organizational Research: Core Methods and Current Challenges. London: Sage 2012.38.

38. Hardcastle MR, Usher KJ, Holmes CA, An overview of structuration theory and its usefulness in nursing research. *Nurs Philos* 2005; 6: 223-234

39. Greenhalgh T, Russell J. Why do evaluations of eHealth programs fail? An alternative set of guiding principles. *PLOS Med*. 2010: 7:e1000360

40. Doolin B. Power and resistance in the implementation of a medical management information system. *Inform Syst J* 2004;14:343–62.

41. Cresswell KM, Worth A, Sheikh A. Integration of a nationally procured electronic health record system into user work practices *BMC Med Inform Decis Mak*. 2012;12:15

42. Sheikh A, Cornford T, Barber N, et al. Implementation and adoption of nationwide electronic health records in secondary care in England: final qualitative results from prospective national evaluation in "early adopter" hospitals. *BMJ*. 2011; 343:d6054

43. Waterson P. Health information technology and sociotechnical systems: A progress report on recent developments within the UK National Health Service (NHS). *Appl Ergon*. 2014; 45: 150-161

44. Daker-White G, Hays R, McSharry J, et al. Blame the patient, blame the doctor? A meta-synthesis in qualitative studies of patient safety in primary care. *PLOS One*. 2015; 10(8), e0128329

For peer review only - http://bmjopen.bmj.com/site/about/guidelines.xhtml

45. Winthereik BR, van der Ploeg I, Berg M. The electronic patient record as a meaningful audit tool: accountability and autonomy in general practitioner work. *Sci Technol Hum Values* 2007;32:6–25.

46. Esmail, A. *Measuring and monitoring safety: a primary care perspective.* London: The Health Foundation 2013.

47. Cresswell KM, Bates DW, Sheikh A. Ten key considerations for the successful implementation and adoption of large-scale health information technology. *J Am Med Inform Assoc.* 2013; 0, 1-5

48. Swinglehurst D, Greenhalgh T, Myall M, et al. Ethnographic study of ICT-supported collaborative work routines in general practice. *BMC Health Serv Res*. 2010;10:348

For peer review only

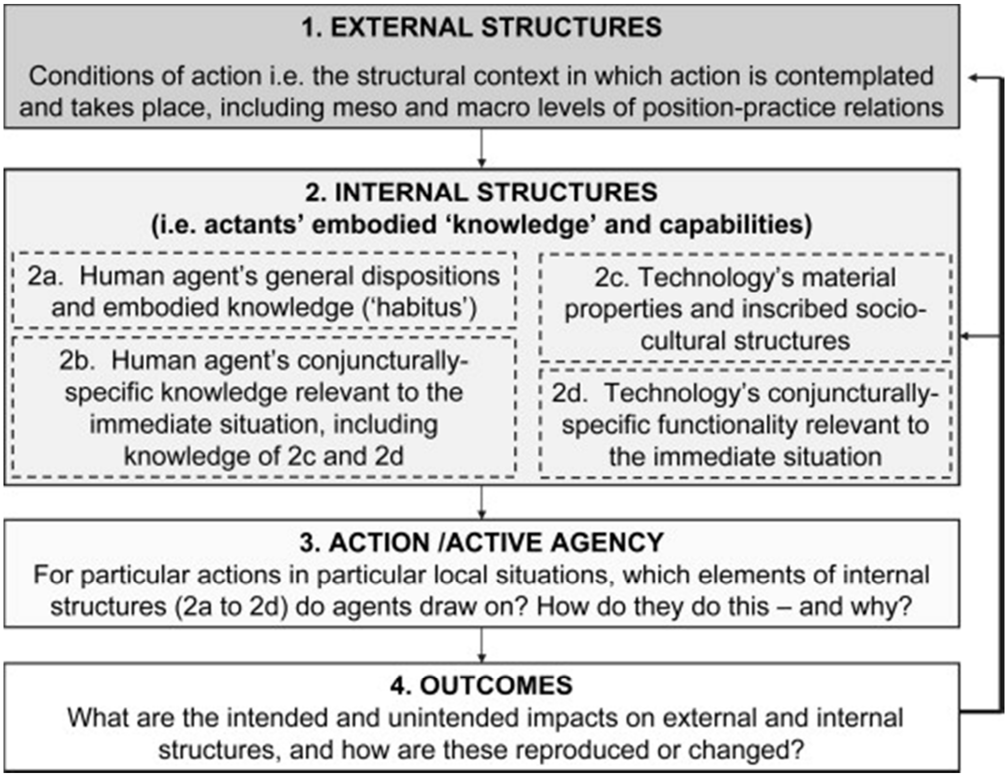

120x92mm (113 x 113 DPI)

Understanding the implementation and adoption of an information technology intervention to support medicines optimisation in primary care: qualitative study using strong structuration theory

COREQ checklist

Note: in order to minimize the length of the manuscript, some of the details on the checklist (marked '*') are not included in the manuscript.

Guide question		*Response*	*Page number in manuscript*
1	Interviewer/facilitator	MJ conducted the interviews. MJ and RLH facilitated the focus groups	9
2	Credentials	MJ holds an MSc in Health Psychology<br>RLH holds a PhD in Medic8ines safety in Primary care	9
3	Occupation	MJ : Research Associate in medication safety;<br>RLH: freelance research pharmacist	9
4	Gender	MJ male; RLH female	9
5	Experience and training	Both researchers have previous experience of undertaking qualitative research in healthcare at PhD and postdoctoral level	9
6	Relationship established	The researchers were not known to the participants prior to the study	9
7	Participant knowledge of the researcher	Participants were made aware of the reasons for doing the research via the information which was sent to the participant prior to the interview	*
8	Researcher characteristics	The researchers had identified the study topic as part of larger programmes of work in their research groups, medication safety in primary care.	9
9	Methodological orientation and theory	Strong structuration theory. The analysis was thematic using template analysis.	5-6, 10
10	Sampling	Individual participants were recruited on a purposive basis via the study CCG or through community pharmacy networks. All participants were chosen to fit the sampling frame (people within the CCG's geographical area who represented the stakeholder groups: pharmacists, doctors, general practice managers and patients)	8-9
11	Method of approach	Participants were approached by telephone or email	9
12	Sample size	19 participants	8-9
13	Non-participation	A number of possible participants were approached but declined to participate. Predominantly this was for reasons of time, workload or lack of use of the system. These included 2 pharmacist technicians, 2 GPs, 2 community pharmacists and 8 general practice managers.	*
14	Setting of data collection	Four interviews were conducted by telephone and one at the CCG offices, the focus groups were conducted at the CCG offices or at a local hotel.	9
15	Presence of non-participants	No non-participants were present	9

For peer review only

16	Description of sample	See Table 1 of the main manuscript	8
17	Interview guide	In the interviews and focus groups we explored experiences of working with the EMOS, perceptions of the system, benefits and drawbacks, the organisational structures and roles required for its use and the circumstances under which it was considered most effective. No pilot testing was undertaken due to the small scale nature of the study, the timescale of the study and the difficulties of recruitment.	9
18	Repeat interviews	None -One GP was interviewed and participated in a focus group	9
19	Audio/visual recording	Audio recording only, with consent from the participant	9
20	Field notes	None	*
21	Duration	The interviews lasted between 20 and 50 mins. The focus groups lasted between 57-112 mins.	9
22	Data saturation	Data collection continued until saturation was reached and no new themes emerged from the interviews and focus groups.	9
23	Transcripts returned	No transcripts were returned to participants	*
24	Number of data coders	MJ coded the data but regular discussions codes were held with all authors. Coding template reviewed by MJ and DLP	10
25	Description of the coding tree	A coding tree description is not given but details on a priori codes is included	10
26	Derivation of themes	A priori thematic codes were applied to the data and new themes emerged from the data. This is described in the analysis section	10
27	Software	QSRNvivo 10 software was utilised to manage the data	10
28	Participant checking	No	*
29	Quotations presented	Please see the results section of the manuscript	10-17
30	Data and findings consistent		10-17
31	Clarity of major themes		10-17
32	Clarity of minor themes		10-17

For peer review only
